# Higher maternal parathyroid hormone concentration at delivery is not associated with smaller newborn size

**DOI:** 10.1530/EC-21-0056

**Published:** 2021-02-23

**Authors:** Huma Qamar, Nandita Perumal, Eszter Papp, Alison D Gernand, Abdullah Al Mahmud, Daniel E Roth

**Affiliations:** 1 Centre for Global Child Health, Hospital for Sick Children, Toronto, Ontario, Canada; 2 Department of Nutritional Sciences, University of Toronto, Toronto, Ontario, Canada; 3 Department of Epidemiology, Dalla Lana School of Public Health, University of Toronto, Toronto, Ontario, Canada; 4 Department of Nutritional Sciences, Pennsylvania State University, University Park, Pennsylvania, USA; 5 Nutrition and Clinical Services Division, International Centre for Diarrhoeal Disease Research (icddr,b), Dhaka, Bangladesh; 6 Department of Paediatrics, Hospital for Sick Children and University of Toronto, Toronto, Ontario, Canada

**Keywords:** parathyroid hormone, pregnancy, fetal growth, intrauterine growth restriction, vitamin D, 25-hydroxyvitamin D, FGF23, small-for-gestational age

## Abstract

Intrauterine growth restriction (IUGR) reflects inadequate growth in-utero and is prevalent in low resource settings. This study aimed to assess the association of maternal delivery parathyroid hormone (PTH) – a regulator of bone turnover and calcium homeostasis – with newborn anthropometry, to identify regulators of PTH, and to delineate pathways by which maternal PTH regulates birth size using path analysis. This was a cross-sectional analysis of data from participants (*n* = 537) enrolled in the Maternal Vitamin D for Infant Growth trial in Dhaka, Bangladesh. Primary exposures were maternal delivery intact PTH (iPTH) or whole PTH (wPTH) and outcomes were gestational age- and sex-standardized z-scores for birth length (LAZ), weight (WAZ), and head circumference (HCAZ). Hypothesized regulators of PTH included calcium and protein intake, vitamin D, magnesium, fibroblast-like growth factor-23 (FGF23), and C-reactive protein. Maternal iPTH was not associated with birth size in linear regression analyses; however, in path analysis models, every SD increase in log(iPTH) was associated with 0.08SD (95% CI: 0.002, 0.162) higher LAZ. In linear regression and path analysis models, wPTH was positively associated with WAZ. Vitamin D suppressed PTH, while FGF23 was positively associated with PTH. In path analysis models, higher magnesium was negatively associated with LAZ; FGF23 was positively associated and protein intake was negatively associated with LAZ, WAZ, and HCAZ. Higher maternal PTH in late pregnancy is unlikely to contribute to IUGR. Future studies should investigate maternal FGF23, magnesium and protein intake as regulators of fetal growth, particularly in settings where food insecurity and IUGR are public health problems.

## Introduction

Intrauterine growth restriction (IUGR), or inadequate fetal growth, is associated with short- and long-term adverse health outcomes (1, 2, 3, 4, 5). Low birthweight (LBW), defined as birthweight less than 2500 g, and small-for-gestational age (SGA), defined as birthweight below the 10^th^ centile of gestational age- and sex-matched healthy reference population, are conventional indicators of IUGR. Length and head circumference at birth are important indicators of fetal skeletal growth and are correlated with indicators of IUGR (6, 7); for example, birth length strongly correlates with fetal femur length based on ultrasound (8). In low- and middle-income countries such as Bangladesh, where LBW and SGA are prevalent (9), reductions in both newborn weight and length are observed (10, 11).

Parathyroid hormone (PTH) is a peptide hormone that primarily regulates calcium homeostasis by binding to the PTH 1 Receptor (PTH1R) (12, 13), and is itself regulated by vitamin D and calcium in feedback loops. Chronic exposure of bones to PTH is generally observed to lead to decreases in bone mineral content (12). Evidence from *in vivo* and clinical studies suggests that parathyroid hormone does not cross the placenta (14, 15). As such, maternal PTH likely regulates bone growth through transplacental calcium flux or other indirect mechanisms.

During pregnancy, the increased demand for calcium to support fetal growth is met by increasing maternal intestinal absorption of calcium from the diet, reabsorption from the kidneys, and resorption of bone (16). In some women, plasma/serum PTH concentrations increase during pregnancy, which may help to mobilize calcium and promote fetal growth in settings of dietary calcium deficits or vitamin D deficiency (15, 17, 18, 19). However, in an American pregnancy cohort, Scholl *et al*. reported that PTH was negatively associated with birth length and head circumference (20). Furthermore, women with elevated PTH and either low maternal dietary calcium intake and/or vitamin D deficiency had a higher risk of SGA and lower birth length and head circumference (20). Studies in Ireland and Norway reported no association between PTH and SGA or birthweight (21); however, an Australian study reported a positive association between maternal PTH concentrations and knee-to-heel length (22). These inconsistent findings and the lack of evidence from low- and middle-income settings with a high prevalence of SGA/LBW warrant further investigation.

To understand the biological mechanisms by which PTH may be associated with fetal growth, regulators of PTH are important to consider. PTH synthesis and activity may be regulated by a limited set of nutritional and endocrine factors. The vitamin D metabolite 1,25-dihydroxyvitamin D directly regulates PTH in the parathyroid through negative feedback loops to suppress circulating concentrations (23, 24). Calcium is a direct suppressor of PTH through the calcium-sensing receptor (25). Fibroblast-like growth factor-23 (FGF23), a marker of bone metabolism, has also been postulated as a regulator of PTH secretion, although the direction of this effect is not yet well characterized (26, 27, 28, 29, 30). Additional factors that may regulate PTH include magnesium and systemic inflammation markers such as C-reactive protein (CRP) (13, 31, 32, 33). These factors may serve as points of intervention, for example, through modifying maternal dietary intake of vitamin D, calcium, or magnesium. However, the relationships between nutritional and endocrine regulators of PTH, and their direct or indirect (i.e. PTH-mediated) associations with newborn size, have not been fully elucidated.

In a population with concurrent high prevalences of IUGR, vitamin D deficiency, and dietary calcium deficits, this study aimed to estimate the associations between maternal PTH concentrations and newborn size at birth, to identify potentially modifiable nutritional and endocrine factors associated with maternal PTH, and to assess the direct and indirect (i.e. PTH-mediated) pathways by which maternal PTH and its regulators may influence fetal growth. We hypothesized that PTH would be negatively associated with birth size due to a higher prevalence of calcium metabolic stress in this population. We also hypothesized that PTH would function as a mediator of the association of upstream regulators of PTH and birth size.

## Methods

### Study design

This was a cross-sectional analysis of data from a subset of participants enrolled in the Maternal Vitamin D for Infant Growth (MDIG) Trial in Dhaka, Bangladesh (Trial Registration Number: NCT01924013, clinicaltrials.gov). The MDIG trial was a double-blind, randomized placebo-controlled trial designed to estimate the dose-ranging effects of prenatal vitamin D supplementation (4200 IU/week, 16,800 IU/week, and 28,000 IU/week) and postpartum supplementation (28,000 IU/week among half the women receiving 28,000 IU/week prenatally), vs placebo on infant length at 1 year of age. Participants enrolled in the MDIG trial were co-supplemented with daily calcium (500 mg) and iron and folic acid (66 mg elemental iron, 350 μg folic acid). Detailed descriptions of the methods and results of the trial are described elsewhere (34, 35). Approval for the MDIG trial and biochemical analyses related to the PTH axis was obtained from research ethics committees at the Hospital for Sick Children and the International Center for Diarrheal Disease Research, Bangladesh (icddr,b). Written informed consent was obtained from all participants for participation and storage of biological specimens.

### Study subjects

Healthy pregnant women attending antenatal visits at the Maternal and Child Health Training Institute (MCHTI) at least 18 years of age and at 17–24 completed weeks of gestation at the time of the visit (based on recalled last menstrual period and ultrasound performed by technicians at MCHTI) were invited to participate in the MDIG trial. Detailed inclusion and exclusion criteria are described elsewhere (34, 35).

A total of 537 mother–infant pairs were selected for this study (Supplementary Fig. 1, see section on supplementary materials given at the end of this article). Participants were selected from a subset of mother-infant pairs in MDIG selected for biochemical analysis based on availability of infant and/or cord blood samples. Additional inclusion criteria for the present study included maternal blood specimens collected at delivery and newborn anthropometry performed within 48 h of birth (*n* = 490). To increase the sample size to describe changes in PTH concentrations from mid-gestation to delivery in the absence of vitamin D supplementation, an additional 47 participants from the placebo group were also included in this study (Supplementary Fig. 1).

## Data collection

### Clinical, sociodemographic and dietary data

Baseline questionnaires at enrollment collected maternal age, gravidity, and socioeconomic status indicators. Questionnaires administered at the delivery visit provided information on sex of the newborn and season of birth. Gestational age at birth was derived using recorded date of birth and recalled last menstrual period and/or baseline ultrasound. A 41-item non-quantitative food frequency questionnaire (FFQ) was administered at baseline to estimate habitual maternal calcium and protein intake (Supplementary Method 1). Calcium intake was expressed in milligrams per day (mg/day) and protein intake was expressed in grams of protein per kilogram body weight per day (g/kg/day).

### Infant anthropometry

Crown-to-heel length, rump-to-knee length (RKL), weight, and head circumference were measured independently by two study personnel within 48 h of birth, based on a predefined protocol (Supplementary Method 2). Length was initially measured using a length board with a counter display and ball bearing mounted sliding footboard (Harpenden infantometer; Holtain, Crymych, UK); however due to frequent decalibration, it was changed to a wooden length board (Infant/Child ShorrBoard; Weigh and Measure, Olney, MD, USA). A total of 128 infants (24%) were measured using the Harpenden length board and 402 infants (76%) were measured using the Shorrboard.

Intergrowth-21st growth standards (36) were used to derive sex- and gestational age-adjusted z-scores for length (LAZ), weight (WAZ), and head circumference (HCAZ).

### Specimen collection and laboratory methods

Maternal specimens were collected and stored using a standard protocol (Supplementary Method 3). Assay information and performance indicators for all lab analyses are recorded in Supplementary Table 1.

Maternal baseline and delivery plasma PTH concentrations were quantified using intact PTH (iPTH) and whole PTH (wPTH) sandwich ELISA kits (Immutopics 60-3100 and 60-3000, respectively, Athens, OH, USA). The iPTH assay measures concentrations of the whole 84 amino acid PTH peptide and long fragments that are missing the first few amino acids from the n-terminus because the epitope for the detection antibody is located in the middle of the peptide. The wPTH assay uses a detection antibody targeting the first four amino acids of the PTH protein, and therefore measures only the whole (bioactive) PTH peptide without any inactive fragments. Although wPTH may provide a more physiologically relevant measure of the active PTH peptide, we also present analyses using iPTH to compare to prior studies of the association of maternal PTH and birth size, all of which used iPTH assays (20, 21, 22, 37).

Maternal serum 25-hydroxyvitamin D (25(OH)D) concentrations were measured using high-performance liquid chromatography-tandem mass spectrometry (LC-MS/MS) following methodology previously described (38). Maternal plasma FGF23 was measured using a second-generation c-terminal FGF23 ELISA. Maternal plasma CRP was measured using a sandwich ELISA and maternal serum magnesium was measured using a quantitative colorimetric assay.

If any biomarker analyses yielded unquantifiable values due to concentrations being below the standard curve detection limit, half the lowest standard of the assay was imputed for analysis (Supplementary Table 1). Values above the upper limit of detection were imputed as the highest standard curve concentration. For CRP, values that were above the upper limit of detection were imputed extrapolating from the standard curve (Supplementary Table 1).

### Data analysis

Participant characteristics and biomarker concentrations were expressed as mean ± s.d., median (25th percentile, 75th percentile), or frequencies and percentages. Maternal PTH, FGF23, CRP, and 25(OH)D were log-transformed due to right-skewing of the data. Participant characteristics were compared between women with baseline iPTH concentrations (17–24 weeks’ gestation) that were above or below the 80th percentile of the iPTH distribution (5.81 pmol/L). Similar to Hemmingway *et al.* (21), we used the 80th percentile of the distribution to define elevated iPTH, as there is no established reference ranges for PTH in pregnancy. Among women randomized to the placebo group, we reported baseline and delivery iPTH and wPTH concentrations, stratified by 25(OH)D status (<30 nmol/L; 30–50 nmol/L; ≥50 nmol/L) and/or calcium intake (above/below the 50th percentile of intake).

To estimate the associations of maternal delivery PTH (iPTH and wPTH) with birth LAZ, WAZ, and HCAZ, we used unadjusted and multivariable-adjusted linear regression models. A modified Poisson regression with robust error variance (39) was used to estimate the association of delivery PTH with the risk of being SGA. Potential confounders selected *a priori* for inclusion in multivariable regression models included magnesium, FGF23, CRP, vitamin D treatment group, maternal age, maternal height, maternal education, asset index (derived from participant ownership of various items (34)), gravidity, gestational age at birth, season of birth, and protein intake. Pre-pregnancy maternal weight/BMI were not available to be included in the multivariable model; however, in sensitivity analyses, we found that including postpartum BMI did not change inferences (data not shown). Models, where RKL was the outcome, were additionally adjusted for sex. Although vitamin D did not significantly affect any newborn size parameters in the main trial analyses (34), vitamin D group was included in all models. All effect estimates were scaled to a 90% increase in iPTH and a 73% increase in wPTH concentrations to reflect a large but biologically plausible difference in PTH concentrations, which corresponds to the observed difference in delivery PTH between the highest-dose vitamin D intervention (28,000 IU/week) and the placebo group in the MDIG cohort (34). Sensitivity and subgroup analyses were conducted to assess the robustness of our findings (Supplementary Method 4).

To identify regulators of PTH, delivery iPTH and wPTH concentrations were each regressed on vitamin D treatment group, magnesium, FGF23, CRP, protein intake, and calcium intake in separate linear regression models. Multivariable models included all proposed regulators of PTH, maternal age, maternal education, asset index and gravidity. In sensitivity analyses, vitamin D treatment group was substituted with 25(OH)D concentrations in multivariable models, and analyses were limited to women randomized to receive placebo (*n* =142).

To test an integrated biological model of the direct and indirect pathways by which delivery PTH and its regulators are associated with birth LAZ, WAZ, and HCAZ, path analysis was used as a method to simultaneously perform mediation analyses (Supplementary Method 5). From the path analysis model, we present direct, indirect (mediated) and total effects, which represented both associations and causal effects (when vitamin D supplementation was the predictor). Maternal calcium intake and delivery CRP concentrations were not included in the final path model as they did not explain a meaningful level of variance in delivery PTH and birth size, and did not improve model fit.

All results were presented as point estimates of the effect size, with 95% CIs and *P*-values. We considered *P*-values less than 0.05 as statistically significant. Data were analyzed with Stata version 15.1 (StataCorp 2017).

## Results

### Maternal and infant characteristics

Participant characteristic and biomarker data, stratified by baseline iPTH concentration (measured at 17–24 weeks of gestation), are summarized in Table 1. With the exception of 25(OH)D concentrations at baseline, delivery iPTH and wPTH, participant characteristics did not differ by baseline iPTH. Participants selected for this sub-study were generally not different compared to the rest of the MDIG cohort; however, a higher proportion of women in this sub-study gave birth by cesarean section, which is likely due to a higher proportion of hospital births in our subsample (Supplementary Table 2). Median iPTH concentration at delivery was 3.3 pmol/L (IQR: 2.1, 5.2) and wPTH concentration at delivery was 2.8 pmol/L (IQR: 1.8, 4.3). In the absence of vitamin D supplementation (i.e. in the placebo group), maternal PTH concentrations significantly increased from enrollment to delivery (Supplementary Fig. 2). Baseline and delivery PTH concentrations among women in the placebo group with enrollment 25(OH)D concentrations greater than 30 nmol/L were lower than in women with 25(OH)D concentrations below 30 nmol/L (Supplementary Table 3). Women with baseline calcium intake above the 50th percentile tended to have lower PTH concentrations at baseline but higher concentrations at delivery compared to women with baseline calcium intake below the 50th percentile (Supplementary Table 3).

**Table 1:** Maternal and infant demographic and clinical characteristics, stratified by baseline (17–24 weeks gestation) maternal iPTH concentrations among mother-infant pairs in a pregnancy cohort in Bangladesh.^a^

	Baseline iPTH < 5.81 pmol/L	Baseline iPTH ≥ 5.81 pmol/L	*P*^b^
Included participants, *n*	426	110	
Maternal Baseline Characteristics			
Age (years), median (min, max)	23 (18, 38)	23 (18, 38)	0.94
Level of education, *n* (%)			0.79
Little to no schooling	154 (36)	38 (35)	
Some or completed secondary education	222 (52)	61 (55)	
Some or completed tertiary education	50 (12)	11 (10)	
Asset index quintiles, *n* (%)^c^			0.56
1 (lowest)	85 (20)	23 (21)	
2	81 (19)	22 (20)	
3	77 (18)	23 (21)	
4	97 (23)	17 (15)	
5 (highest)	85 (20)	25 (23)	
Gravidity, median (min, max)	2 (1, 7)	2 (1, 9)	0.75
Height (cm), mean ± s.d.	151.0 ± 5.5	150.7 ± 5.5	0.60
Estimated dietary calcium intake (mg/day), mean ± s.d.	983.5 ± 279.2	989.2 ± 245.0	0.85
Estimated dietary protein intake (g/kg/day), mean ± s.d.	0.8 ± 0.4	0.9 ± 0.3	0.26
Treatment group			0.26
Placebo	105 (25)	37 (34)	
4200 IU/week	77 (18)	18 (16)	
16,800 IU/week	91 (21)	18 (16)	
28,000 IU/week	153 (36)	37 (34)	
Delivery characteristics			
Gestational age at birth (weeks), median (min, max)^d^	39.2 (33, 43)	39.1 (35, 42)	0.90
Preterm (<37 weeks), *n* (%)	30 (7.0)	3 (2.7)	0.09
Cesarean section, *n* (%)^d^	257 (60)	73 (66)	0.25
Girls, *n* (%)^d^	214 (50)	50 (45)	0.37
Season of birth, *n* (%)			0.29
Spring (March–May)	70 (16)	14 (13)	
Summer (June–August)	108 (25)	21 (19)	
Fall (September–November)	137 (32)	43 (39)	
Winter (December–February)	111 (26)	32 (29)	
Gestational age/sex-standardized growth parameter, mean ± s.d.			
WAZ at birth^d^	−1.18 ± 0.85	−1.15 ± 0.85	0.70
LAZ at birth^e^	−0.90 ± 0.99	−0.88 ± 1.02	0.88
HCAZ at birth^f^	−0.61 ± 0.95	−0.60 ± 0.98	0.97
SGA, *n* (%)^d^	199 (47)	48 (44)	0.54
Maternal baseline biochemistry			
iPTH (pmol/L), median (IQR)	3.2 (2.4–4.3)	7.3 (6.6–9.7)	<0.001
Detectable iPTH, *n* (%)	394 (92)	110 (100)	
wPTH (pmol/L), median (IQR)^g^	2.8 (2.1–3.9)	5.8 (4.2–7.7)	<0.001
Detectable wPTH, *n* (%)	344 (87)	90 (96)	
25(OH)D (nmol/L), mean ± s.d.^h^	28.9 ± 14.4	22.4 ± 13.2	<0.001
Maternal delivery biochemistry			
iPTH (pmol/L), median (IQR)	3.0 (1.9–4.5)	4.9 (3.0–7.8)	<0.001
Detectable iPTH, *n* (%)	346 (81)	104 (95)	
wPTH (pmol/L), median (IQR)^g^	2.5 (1.7–3.7)	4.3 (2.6–6.5)	<0.001
Detectable wPTH, *n* (%)	309 (78)	88 (94)	
25(OH)D (nmol/L), mean ± s.d.^i^	85.1 ± 40.8	76.6 ± 42.4	0.07
Magnesium (mmol/L), mean ± s.d.^j^	0.8 ± 0.1	0.8 ± 0.2	0.84
FGF23 (RU/mL), median (IQR)	116.4 (74.3–201.1)	124.7 (85.0–225.2)	0.21
CRP (mg/L), median (IQR)^d^	8.3 (4.0–18.8)	11.2 (4.0–22.7)	0.14

^a^Baseline iPTH was split at the 80th percentile of the distribution (5.81 pmol/L). Baseline PTH data was not available for 1 woman in the study (randomized to 4200 IU/week treatment group). ^b^*P*-values presented are from Chi-square or Fischer Exact Tests for categorical variables and from ANOVA or Kruskal–Wallis tests for continuous variables. ^c^*n* = 425 among women with baseline iPTH < 5.81 pmol/L. ^d^*n* = 424 among women with baseline iPTH < 5.81 pmol/L. ^e^*n* = 421 among women with baseline iPTH < 5.81 pmol/L and *n* = 108 among women with baseline iPTH ≥ 5.81 pmol/L. ^f^*n* = 424 among women with baseline iPTH < 5.81 pmol/L and *n* = 109 among women with baseline iPTH ≥ 5.81 pmol/L. ^g^*n* = 397 among women with baseline iPTH < 5.81 pmol/L and *n* = 94 among women with baseline iPTH ≥ 5.81 pmol/L. ^h^*n* = 423 among women with baseline iPTH < 5.81 pmol/L. ^i^*n* = 400 among women with baseline iPTH < 5.81 pmol/L and *n* = 96 among women with baseline iPTH ≥ 5.81 pmol/L. ^j^*n* = 398 among women with baseline iPTH < 5.81 pmol/L and *n* = 96 among women with baseline iPTH ≥ 5.81 pmol/L.

### Association of maternal PTH and birth size

iPTH at delivery was significantly positively associated with LAZ, WAZ, and HCAZ in unadjusted linear regression models, but all estimates were attenuated and non-significant in multivariable linear regression models (Table 2 and Supplementary Tables 4, 5, 6, 7, 8). In unadjusted and multivariable models, wPTH at delivery was positively associated with WAZ and was associated with a lower risk of being born SGA (Table 2).

**Table 2:** Differences in birth size parameters for a 90% increase in maternal delivery intact PTH (iPTH) or 73% increase in maternal whole PTH (wPTH) concentrations among mother–infant pairs from a birth cohort in Dhaka, Bangladesh.

	Unadjusted models				Multivariable model^a^			
	*n*	Difference in size/RR^b^	95% CI	*P*^c^	*n*	Difference in size/RR^b^	95% CI	*P*^c^
iPTH								
LAZ	530	0.091	0.020, 0.161	0.012	487	0.064	−0.011, 0.139	0.09
WAZ	535	0.070	0.010, 0.129	0.022	490	0.056	−0.007, 0.118	0.08
HCAZ	534	0.082	0.015, 0.149	0.017	489	0.047	−0.027, 0.121	0.21
RKL	532	0.004	−0.050, 0.058	0.89	487	−0.004	−0.062, 0.053	0.88
SGA	535	0.92	0.85, 0.99	0.023	490	0.94	0.87, 1.02	0.17
wPTH								
LAZ	486	0.033	−0.033, 0.099	0.32	479	0.018	−0.048, 0.084	0.60
WAZ	490	0.070	0.014, 0.125	0.014	482	0.057	0.003, 0.112	0.040
HCAZ	489	0.089	0.028, 0.151	0.004	481	0.061	−0.003, 0.125	0.06
RKL	487	−0.014	−0.064, 0.036	0.59	479	−0.012	−0.062, 0.038	0.64
SGA	490	0.91	0.85, 0.97	0.004	482	0.92	0.86, 0.99	0.024

^a^Multivariable linear or Poisson regression models with robust error variance adjusted for: maternal log PTH, maternal magnesium, maternal log FGF23, maternal log CRP, vitamin D supplementation group, relative protein intake, maternal age, maternal height, maternal education, gravidity, gestational age at birth and season of birth. For models where RKL was the outcome, we additionally adjusted for sex. ^b^Difference in size presented for LAZ, WAZ, HCAZ, and RKL. Relative risk presented for SGA. Effect sizes are for a 90 and 73% increase in iPTH and wPTH concentrations, respectively, which reflects a large but plausible difference in PTH concentrations corresponding to the observed effect of high dose vitamin D supplementation (28,000 IU/week) on PTH, vs placebo. ^c^*P* < 0.05 considered significant. HCAZ, head circumference-for-gestational age z-scores; LAZ, length-for-gestational age z-scores; RKL, rump-to-knee length; SGA, small-for-gestational age; WAZ, weight-for-gestational age z-scores.

There was no significant association between iPTH and birth size in any sensitivity or subgroup analyses (data not shown). Excluding infants who were measured using the Harpenden length board where LAZ was the outcome did not change inferences.

wPTH was not associated with LAZ, WAZ and HCAZ in sensitivity and subgroup analyses, although effect estimates were all positive. Similarly, there were no associations between wPTH and SGA in multivariable subgroup analyses restricted to women randomized to the placebo group, mother-infant pairs with detectable wPTH, boys, and girls. There was, however, a positive association between wPTH and SGA in models adjusting for 25(OH)D in place of vitamin D treatment group; in models restricted to term born infants only; and in models restricted to infants with anthropometry collected within 24 h of birth (data not shown).

Among covariates included in multivariable models, maternal protein intake was negatively associated with birth LAZ, WAZ, and HCAZ, whereas FGF23 concentrations showed positive associations with LAZ, WAZ, HCAZ, and RKL (Supplementary Tables 4, 5, 6, 7 and 8). Among mother-infant pairs randomized to the placebo group, maternal baseline PTH was not associated with newborn size (Supplementary Table 9).

### Association of nutritional and endocrine factors with PTH concentrations

**Figure 1:**
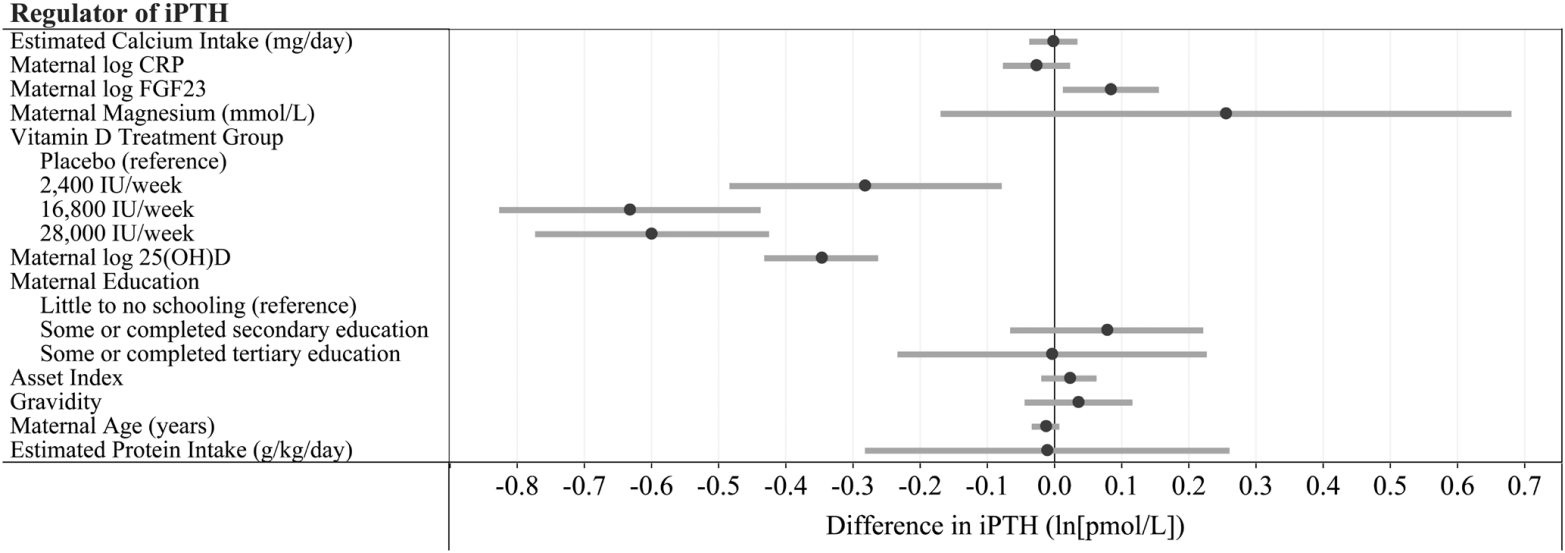
Multivariable linear regression analysis of the association of maternal calcium intake, protein intake, delivery magnesium concentrations, delivery fibroblast-like growth factor 23 (FGF23), delivery C-reactive protein (CRP), delivery 25-hydroxyvitamin D (25(OH)D) and prenatal vitamin D supplementation with maternal delivery log intact PTH (iPTH) concentrations. The multivariable model adjusted for each of the regulators as well as baseline protein intake, maternal education, asset index, gravidity, and maternal age at enrollment. Effect estimates represent changes in log iPTH for: a 100 mg/day increase in calcium intake; a unit increase in log CRP, FGF23 and 25(OH)D; and a 1 mmol/L increase in magnesium. Black dots represent the regression coefficient from multivariable linear regression models, gray bars represent the 95% CIs of the regression coefficient. The estimate for maternal log 25(OH)D was estimated in a separate model, replacing vitamin D treatment group.

We used linear regression analyses to assess the association between hypothesized nutritional and endocrine factors and maternal PTH concentrations at delivery. Vitamin D supplementation suppressed iPTH and wPTH concentrations at delivery in a dose-dependent manner (Fig. 1 and Supplementary Tables 10, 11). Similarly, we observed significant negative associations between delivery 25(OH)D and delivery PTH. Serum magnesium and plasma FGF23 were positively associated with iPTH and wPTH in unadjusted models. In multivariable models, delivery FGF23 remained significantly associated with delivery iPTH and wPTH; however, magnesium only remained associated with wPTH. The association between magnesium and iPTH was primarily attenuated by inclusion of vitamin D supplementation in the model. Daily calcium intake and CRP concentrations were not found to be associated with delivery PTH.

### Direct and indirect associations of PTH and its regulators with newborn size using path analysis

**Figure 2:**
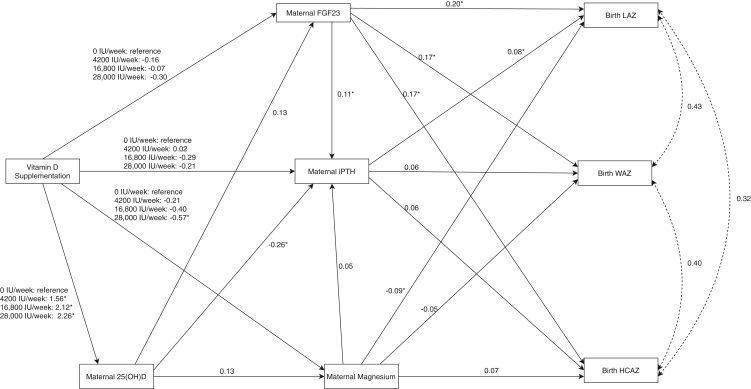
Path diagram showing the direct and indirect pathways by which maternal delivery intact parathyroid hormone (iPTH) and its associated nutritional and endocrine factors are associated with newborn size at birth. Each of the arrows denote tested paths and are labeled with the standardized path coefficients, which represent the direct effect of the explanatory variable on the dependent variable. Double headed arrows represent covariances between variables. Asterisks denote statistically significant path coefficients (*P* < 0.05). Confounders included in the path analysis model are not shown in this figure.

The final path models demonstrated very good model fit (iPTH model: RMSEA < 0.001, TLI = 1.035; wPTH model: RMSEA < 0.001, TLI = 1.028). Similar to linear regression models, vitamin D supplementation suppressed PTH at delivery in a dose-dependent manner; the lowest dose at which maximal suppression was achieved was the 16,800 IU/week group, with no additional suppression of PTH in the 28,000 IU/week group (Supplementary Tables 12 and 13). There was a significant negative direct and total effect of delivery 25(OH)D on PTH concentrations. The direct effect of vitamin D supplementation was not significant; however, the indirect effect of vitamin D supplementation was statistically significant, indicating the effect of prenatal vitamin D supplementation on delivery PTH was substantially mediated by delivery 25(OH)D (Fig. 2 and Supplementary Table 12). The indirect effect of vitamin D supplementation on iPTH in the 16,800 IU/week group and 28,000 IU/week group (compared to placebo) accounted for 65%, and 74% of the total effect, respectively.

Maternal delivery FGF23 was significantly associated with delivery iPTH and wPTH concentrations. Magnesium had a positive direct effect on wPTH. The highest dose of vitamin D supplementation suppressed delivery magnesium compared to placebo, with no significant effect at intermediate doses, although effect estimates indicated a dose-dependent response (Fig. 2).

iPTH at delivery was positively associated with LAZ at birth (direct and total effects) (Fig. 2 and Supplementary Table 12). wPTH at delivery was positively associated with birth WAZ (Supplementary Table 13). Magnesium concentrations were negatively associated with LAZ and delivery FGF23 was positively associated with LAZ, WAZ, and HCAZ (Fig. 2 and Supplementary Table 12). The indirect effect of FGF23, mediated by iPTH, accounted for only 4.2, 3.9, and 3.8% of the total effect of FGF23 on LAZ, WAZ, and HCAZ, respectively.

Analyses assessing whether supplementary vitamin D or tertiles of usual dietary calcium intake modified the iPTH-LAZ association revealed no vitamin D-iPTH or calcium-iPTH interactions relevant to the association of iPTH and infant length (Supplementary Fig. 3). Similarly, there was no vitamin D-wPTH or calcium-wPTH interactions relevant to the association of wPTH and infant weight (Supplementary Fig. 4).

## Discussion

In a Bangladeshi cohort with a high prevalence of SGA, low dietary calcium intake, and high prevalence of vitamin D deficiency, we did not find strong evidence to support an important role for PTH in the regulation of fetal growth. Although there was a positive association between maternal delivery iPTH concentrations and birth length in a path analysis model, the effect sizes were small, and inferences were not supported by models adjusted for confounders. Similarly, while wPTH was positively associated with birthweight in path analysis and multivariable linear regression models, overall effect sizes were small. Effect estimates across all models assessing the association of iPTH and wPTH with birth anthropometry were positive, which was contrary to our hypothesized negative effect of maternal PTH on fetal growth. These results are generally consistent with the previously reported null effect of vitamin D supplementation on birth size in the main trial report (34).

Morley and colleagues previously reported a positive association between late-pregnancy iPTH and birthweight in an Australian cohort (22). Although our estimates for the association of iPTH and wPTH with birthweight were similar, the effect size for the wPTH-birthweight association was more precise (i.e. the standard errors of the estimates were smaller). In contrast, in a study conducted in a cohort of Pakistani women living in Oslo, delivery iPTH was negatively associated with birth length; however, this study was relatively small (N=30) and did not account for confounders (37). In a US pregnancy cohort, there were negative associations of early gestational iPTH (13.8 ± 5.6 weeks of gestation) with birth length and head circumference, and women who experienced ‘calcium metabolic stress’ (defined as insufficient calcium and/or vitamin D intake accompanied by elevated PTH) had a relatively increased risk of SGA birth, lower birth length, and lower head circumference (20). However, these findings contrast observations from a cohort of Irish pregnant women in whom PTH in early pregnancy and calcium metabolic stress at <17 weeks were not associated with SGA, after adjustment for confounders (21).

The timing of PTH measurement during pregnancy may partly explain inconsistent findings across these studies. We and others have observed an increase in PTH concentrations particularly in the third trimester of pregnancy (15, 17, 18, 19, 40). As transplacental calcium transfer occurs to a large extent during the third trimester (16), the increase in PTH may reflect an appropriate physiological response to the demands of fetal skeletal growth. We speculate that higher PTH concentrations early in pregnancy, especially in the context of vitamin D deficiency, is a marker of a suboptimal environment for fetal growth, whereas an elevation in PTH later in pregnancy indicates an appropriate homeostatic response aimed at mobilizing maternal calcium stores to support fetal skeletal growth. We could not corroborate this hypothesis in the present cohort, as there was no association of baseline PTH (17–24 weeks’ gestation) with birth size in sensitivity analyses; this may in part be because baseline PTH in our cohort was mid-gestational, and therefore considerably later in pregnancy than in the study by Scholl and colleagues (20). In addition, due to relatively small sample sizes, we were not able to assess the association of birth size with calcium metabolic stress at delivery as defined by Scholl* et al.* (20). However, we did assess whether vitamin D treatment group or calcium intake (defined as tertiles of intake) modified the iPTH-birth length and wPTH-birthweight associations, and did not find any significant interactions. Therefore, at delivery, the lack of strong association between PTH and infant length and birthweight was similar across varying levels of vitamin D and calcium intake.

We found that the suppressive effect of prenatal vitamin D supplementation on PTH was largely mediated by 25(OH)D. If the observed positive association between iPTH and infant length, and between wPTH and infant weight in our path analysis models are true effects, then we would expect a negative effect of vitamin D supplementation on birth size. This is because the PTH-mediated effect of vitamin D supplementation on birth size is by definition the mathematical product of the direct effects of vitamin D on PTH and of PTH on birth size. Consistent with this notion, the observed indirect effect of vitamin D on birth size was negative, although effect estimates were small and not significant. In the MDIG trial, prenatal vitamin D supplementation indicated very small non-significant total negative effects on birth LAZ, WAZ, and HCAZ, compared to placebo (34). This may suggest that vitamin D is detrimental to fetal growth; however, contrary to this finding, a recent umbrella review of systematic reviews of prenatal vitamin D supplementation found no evidence for an effect of vitamin D on low birthweight and a protective effect on SGA, although quality of evidence was low (41).

Vitamin D also suppressed magnesium concentrations at the highest dose, with an apparent dose-dependent effect. Few studies have reported on the causal effect of vitamin D supplementation on magnesium concentrations. One study conducted in Iran found no effect of vitamin D supplementation (single 50,000 IU bolus dose over 8 weeks) vs placebo on magnesium concentrations among hypertensive patients with vitamin D deficiency (42). Similar null effects of vitamin D supplementation (80 µg/day) on magnesium were noted among obese patients after surgery (43). These studies assessed doses lower than the highest dose group in the MDIG trial, which was the only group in which we observed a significant effect of vitamin D. Magnesium was positively associated with wPTH in multivariable models and was also negatively associated with birth length. The negative association between magnesium and birth length did not appear to be mediated by PTH. Furthermore, the magnesium-mediated indirect effect of vitamin D on birth length was small, but positive (i.e. opposite the negative indirect effect of vitamin D on birth size mediated by PTH). It is unclear why we observed a negative association between maternal magnesium and birth length. One clinical observational study in Ireland reported a positive association between third trimester magnesium intake and birth length, while another study in Poland found no association between placental magnesium and birth size (44, 45). Importantly, our observed effect size of the magnesium-birth length relationship was relatively small and we speculate that there may be other unmeasured pathways that confounded the magnesium-birth length association.

Confirming previous *in vivo* and clinical studies of patients with renal insufficiency (27, 28, 46), we observed a positive association between FGF23 and PTH. Due to the cross-sectional nature of this analysis, we cannot conclude whether FGF23 was a regulator of PTH or vice versa; however, if PTH was a regulator of FGF23, we would have expected a total effect of vitamin D supplementation on FGF23, which was not observed. This null effect is consistent with findings of* post-hoc* analyses from another randomized controlled trial of Austrian hypertensive patients, in whom vitamin D3 supplementation had no effect on FGF23 after adjusting for baseline FGF23 (47). Therefore, although the estimated association between FGF23 and PTH was based on cross-sectional data, the observed null effect of vitamin D on FGF23 suggest that FGF23 is a regulator of PTH.

We observed a robust positive association between FGF23 and birth length, weight, and head circumference as well as an association of higher FGF23 with lower risk of being born SGA. These associations were independent of PTH, and the effect sizes were important. For example, the magnitude of the association for a 1 SD increase in log FGF23 with birth length was almost three-quarters the magnitude of association for a 1 SD increase in maternal height, which is known to correlate strongly with birth length. To our knowledge, no study has directly reported on the association of maternal FGF23 with birth anthropometry. FGF23 may be implicated in fetal long bone growth by promoting hypertrophy and mineralization of chondrocytes (48, 49, 50). However, maternal FGF23 likely does not cross the placenta (51), so any effects it may have on the growing fetus would be through mediated pathways, possibly through its regulatory effects on phosphorous (52).

We did not find strong evidence to support roles for habitual calcium or protein intakes in the regulation of PTH activity in late pregnancy. The lack of apparent effect of calcium intake on maternal PTH concentrations was in line with findings from a cohort of pregnant women in Ireland (53). Although we did not expect protein intake to be a major determinant of PTH, the negative association between estimated protein intake and birth size was consistent with evidence from previous observational studies and trials (54, 55, 56, 57, 58, 59). The association between maternal protein intake and birth size may be U-shaped such that protein intake that is too high or too low could be detrimental to the growing fetus (55), but we did not observe such a U-shaped association of protein intake with birth size.

A strength of the study was the measurement of wPTH in addition to the conventional analyte, iPTH; all previously published observational studies assessing PTH and birth size used iPTH assays (20, 21, 22, 37). Because iPTH assays measure the whole PTH peptide as well as fragments that are missing the active n-terminus and thus unable to bind the PTH1R, we had expected associations to be stronger with wPTH given that it is a more specific measure of the bioactive PTH peptide (60). Effect estimates for wPTH and birth length were smaller than the corresponding estimate for iPTH, although associations with WAZ were slightly stronger for wPTH. Associations of PTH with hypothesized regulators were also not consistently more evident using wPTH, with the exception of its association with serum magnesium. The iPTH and wPTH assays were from the same manufacturer and had comparable inter- and intra-assay CVs, so the similar findings do not seem attributable to wPTH assay performance. Despite our expectations, measuring wPTH did not appear to contribute additional insights beyond what was observed from measuring iPTH.

Our study had limitations that are important to acknowledge. The FFQ utilized in this study was non-quantitative and not previously validated, which may have reduced the accuracy of estimated total dietary calcium and protein intake. Women in the MDIG trial were co-supplemented with calcium which may have mitigated any association of calcium intake with PTH concentrations. Although many of the analyses were based on a cross-sectional observational design (e.g. variables simultaneously measured at birth), we leveraged the randomized placebo-controlled trial design of the MDIG trial to estimate causal effects of vitamin D supplementation on PTH, magnesium, and FGF23. The mediation analyses were grounded in the literature and specific *a priori* hypotheses; therefore, while we are unable to make causal inferences, this study provides important insights into potential mechanisms that influence fetal growth and novel candidate biomarkers that should be further explored (e.g. FGF23). We were also unable to assess associations of venous cord PTH in this study, owing to large proportions of undetectable concentrations (>90%), which would have allowed us to measure the direct association of PTH in the fetal circulation with fetal growth. Lastly, we recognize the potential for selection bias in our analysis, since this study sample was chosen from an already selected group of participants in a randomized controlled trial.

In conclusion, maternal delivery PTH is not a biomarker of IUGR, and elevated maternal PTH at delivery does not appear to impair fetal skeletal growth in a population with a high prevalence of maternal undernutrition and IUGR. Future studies should aim to further examine the potential role of maternal FGF23, magnesium and protein intake in pregnancy on fetal growth.

## Supplementary Material

Supplementary Methods

Supplementary Table 1. Assay information and performance indicators for biochemical measurements.

Supplementary Table 2. Maternal and infant demographic and clinical information of participants in the MDIG trial cohort who were included in this study, compared to those participants who were not included.

Supplementary Table 3. Baseline and delivery iPTH and wPTH concentrations, stratified by vitamin D status and calcium intake among women randomized to the placebo group.

Supplementary Table 4. Multivariable linear regression analysis of the association of maternal delivery iPTH concentrations with infant length-for-age Z scores (LAZ) at birth.

Supplementary Table 5. Multivariable linear regression analysis of the association of maternal delivery iPTH concentrations with infant weight-for-age Z-scores (WAZ) at birth.

Supplementary Table 6. Multivariable linear regression analysis of the association of maternal delivery iPTH concentrations with infant head circumference-for-age Z-scores (HCAZ) at birth.

Supplementary Table 7. Multivariable linear regression analysis of the association of maternal delivery iPTH concentrations with rump-to-knee length (RKL).

Supplementary Table 8. Multivariable modified poisson regression with robust error variance of the association of maternal delivery iPTH concentrations with risk of small-for-gestational age (SGA)

Supplementary Table 9. Multivariable linear regression analysis of the association of maternal iPTH or wPTH concentrations at enrollment (17-24 weeks of gestation) among women randomized to the placebo group with infant LAZ, WAZ, HCAZ, and RKL at birtha.

Supplementary Table 10. Multivariable linear regression analysis of the association of maternal calcium and protein intake, magnesium concentrations, fibroblast like growth factor 23 (FGF23), C-reactive protein (CRP), 25-hydroxyvitamin D (25(OH)D) and vitamin D supplementation with maternal delivery log intact PTH (iPTH) concentrations

Supplementary Table 11. Multivariable linear regression analysis of the association of maternal calcium and protein intake, magnesium concentrations, fibroblast like growth factor 23 (FGF23), C-reactive protein (CRP), 25-hydroxyvitamin D (25(OH)D) and vitamin D supplementation with maternal delivery log whole PTH (wPTH) concentrations.

Supplementary Table 12. Standardized direct and indirect effects of the predictors of intact PTH (iPTH), birth length-for-age z-score (LAZ), birth weight-for-age z-score (WAZ), and birth head circumference-for-age z-score (HCAZ) (n=481)a

Supplementary Table 13. Standardized direct and indirect effects of the predictors of whole PTH (wPTH), birth length-for-age z-score (LAZ), birth weight-for-age z-score (WAZ), and birth head circumference-for-age z-score (HCAZ) (n=473)a

Supplementary Figure 1. Selection criteria for samples eligible for analysis based on the availability of infant samples.

Supplementary Figure 2. Comparison of PTH concentrations at mid-gestation and delivery among women randomized to the placebo group. A) Maternal iPTH concentrations (n=142) at mid-gestation (geometric mean: 3.44 pmol/L [95% CI: 3.04, 3.90]) and delivery (geometric mean: 4.9 pmol/L [95% CI: 4.35, 5.46]); B) Maternal wPTH concentrations (n=97) at mid-gestation (geometric mean: 2.57 pmol/L [95% CI: 2.22, 2.96]) and delivery (geometric mean: 3.74 pmol/L [95% CI: 3.22, 4.35]). The black diamond denotes the geometric means. Coloured lines represent change in PTH concentrations for 10 randomly selected participants. Delivery iPTH and wPTH concentrations were both significantly higher than baseline PTH concentrations, based on paired t-tests of natural log-transformed PTH concentrations (p<0.001). Mid-gestation blood samples were collected between 17 and 24 weeks of gestation (median: 21 weeks), and delivery samples were collected within -19 to 4 days of delivery (median: 0 days). The cluster of data at the bottom of the graph are those women for whom iPTH or wPTH was below the respective lower limit of quantification.

Supplementary Figure 3. Lowess curves of the association between maternal iPTH and length-for-gestational age z-scores (LAZ), stratified by (A) tertiles of estimated calcium intake and (B) vitamin D supplementation. There was no significant interaction between vitamin D and iPTH and calcium intake and iPTH (p>0.05) in models assessing the association between iPTH and LAZ at birth. The large concentration of points at the lower end of the iPTH distribution were below the lower limit of quantification (LOQ) and therefore, imputed as half the lower LOQ.

Supplementary Figure 4. Lowess curves of the association between maternal wPTH and weight-for-gestational age z-scores (WAZ), stratified by (A) tertiles of estimated calcium intake and (B) vitamin D supplementation. There was no significant interaction between vitamin D and wPTH and calcium intake and wPTH (p>0.05) in models assessing the association between wPTH and WAZ at birth. The large concentration of points at the lower end of the wPTH distribution were below the lower limit of quantification (LOQ) and therefore, imputed as half the lower LOQ.

## Declaration of interest

The authors declare that there is no conflict of interest that could be perceived as prejudicing the impartiality of the research reported.

## Funding

The MDIG trial was funded by the Bill and Melinda Gates Foundation (OPP1066764).

## Author contribution statement

H Q, D E R, A D G, and N P designed research; H Q, D E R, E P, and A A M conducted research; H Q analyzed data; and H Q, and D E R wrote the paper. H Q and D E R had primary responsibility for final content. All authors read and approved the final manuscript.

## Acknowledgements

The authors wish to thank all MDIG trial participants, personnel and co-investigators; Dr Robert Bandsma at the Centre for Global Child Health (CGCH), The Hospital for Sick Children (Toronto, Canada) for sharing laboratory resources for this project; Akpevwe Onoyovwi at the CGCH, The Hospital for Sick Children (Toronto, Canada) for assistance with magnesium measurements; and Hayley Craig-Barnes and Ashley St. Pierre of the Analytical Facility for Bioactive Molecules (AFBM), The Hospital for Sick Children (Toronto, Canada) for assistance with 25-hydroxyvitamin D, CRP, and FGF23 measurements. The MDIG trial was funded by the Bill and Melinda Gates foundation.
