# Supplementary material for: Higher maternal parathyroid hormone concentration at delivery is not associated with smaller newborn size
Source: Endocr Connect. 2021 Feb 23;10(3):345–57. doi: 10.1530/EC-21-0056 (PMC8052570; doi:10.1530/EC-21-0056)
Supplement: Supplementary Methods [file supplementary_material.pdf]

# Supplementary Methods

## **Method 1.** Derivation of calcium and protein intake

Calcium and protein intake were derived from a 41-item, non-quantitative food frequency questionnaire (FFQ). The FFQ was designed to estimate relative calcium, phosphorous and phytate intakes in the preceding month. Portion size and calcium content per portion for each item were assumed to be constant across women. Portion sizes were determined based on the logical typical portion size for that food item. We used a combination of the Dietary Guidelines for Bangladesh, the Dietary Guidelines for Indians, and the Canadian Nutrient File to determine portion sizes. A combination of sources was used to fill gaps in available data in the Bangladeshi guidelines. The product of estimated portion size and calcium content per portion was used to weight each food item in the questionnaire; this was the calcium content per portion size. Total daily calcium intake in milligrams per day was then estimated by summing up the mathematical products of reported frequency of intake per day with calcium content per portion for all items on the FFQ. Women in the MDIG trial were also supplemented with 500 mg of elemental calcium daily; therefore, we added to the total calcium intake the product of 500 mg and the percent adherence to vitamin D supplementation in the prenatal period as a proxy of adherence to calcium supplementation, as the latter was not actively monitored. A similar approach was used to derive relative protein intake. Protein intake was standardized to maternal weight in kilograms and expressed as grams of protein per kilogram body weight per day.

## **Method 2.** Infant anthropometric data collection procedures

Crown-to-heel length, rump-to-knee length (RKL), weight, and head circumference were measured independently by two study personnel within 48 hours of birth, based on a predefined protocol (Supplementary Appendix, Method 2). If paired measurements differed by more than a threshold value (7 mm for crown-heel and rump-to-knee length, 50 g for weight, 5 mm for head circumference), a second set of measurements was completed and compared. With the exception of weight, a third set of measurements was obtained if measurements differed by more than the defined threshold. The mean of accepted pairs was used for analyses. RKL and head circumference were measured using a tape measure, and weight using a digital scale (seca 334; Seca, Hamburg, Germany). Length was initially measured using a length board with a counter display and ball bearing mounted sliding footboard (Harpenden infantometer; Holtain, Crymych, UK); however due to frequent decalibration, it was changed to a wooden length board (Infant/Child ShorrBoard; Weigh and Measure, Olney, MD, USA).

## **Method 3.** Specimen collection and storage

Maternal blood samples were collected by a trained phlebotomist at delivery, and processed immediately to generate serum and plasma aliquots. The majority of delivery samples (92%) were collected within one day of delivery. Samples were stored at icddr,b at -70°C until they were shipped on dry ice to the Hospital for Sick Children in Toronto where they were stored at -80°C until batched analysis.

## **Method 4.** Sensitivity and subgroup analyses to assess the association of maternal delivery PTH with birth size

To explore confounding by vitamin D status rather than Supplementary intake, vitamin D treatment group was replaced with serum 25(OH)D concentrations in sensitivity analyses. We also ran all models using raw length, weight, and head circumference instead of z-scores; these models adjusted for sex in addition to all covariates included in the z-score models. Sensitivity analyses were limited to infants born at term (≥ 37 weeks' gestation) (n= 504), infants with birth size measured within 24 hours of birth (n=483), women randomized to receive placebo (n=142), and women with detectable PTH concentrations (n=451). Among women randomized to receive placebo, we also assessed the association of baseline PTH (17-24 weeks of

gestation) with infant size at birth; these models adjusted for the same demographic characteristics as the analysis using delivery data as well as baseline biochemistry (25(OH)D, FGF23, and CRP). As a sensitivity analysis, we also assessed the association of PTH with infant length, excluding Harpenden length board measurements. To assess whether vitamin D supplementation or calcium intake modified significant associations of delivery PTH with infant size at birth, we tested interactions between PTH and vitamin D supplementation, and PTH and calcium intake tertile.

## **Method 5.** Description of path analysis model

We used path analysis to assess the direct and indirect pathways by which PTH is associated with LAZ, WAZ, and HCAZ. A conceptual model was developed *a priori* as a directed acyclic graph to guide the initial model. We allowed for covariances between the anthropometric outcomes to account for the high correlation between newborn anthropometric measures. Multicollinearity was assessed using bivariate correlations between explanatory variables and generating the tolerance statistic and variance inflation factor (VIF), where a tolerance statistic less than 0.2 or VIF greater than 10 was considered evidence of collinearity. Decisions to drop collinear variables were driven by biological significance. Multivariate normality was assessed and variables were transformed as appropriate to attain normality. We allowed for covariances between the anthropometric outcomes to account for the high correlation between newborn anthropometric measures. The absolute fit of the path model was tested using the root mean square error of approximation (RMSEA) statistic, where a $RMSEA<0.1$ was considered good fit and $RMSEA<0.05$ was considered a very good fit. As a test of incremental fit, we used the Tucker Lewis Index (TLI) to compare our model to a competing model, with a $TLI \geq 0.95$ indicating good model fit.
