# Supplementary material for: Higher maternal parathyroid hormone concentration at delivery is not associated with smaller newborn size
Source: Endocr Connect. 2021 Feb 23;10(3):345–57. doi: 10.1530/EC-21-0056 (PMC8052570; doi:10.1530/EC-21-0056)
Supplement: Supplementary Table 1. Assay information and performance indicators for biochemical measurements. [file supplementary_table_1.pdf]

Supplementary Table 1. Assay information and performance indicators for biochemical measurements.

**Biochemical measure**	**Biological matrix**	**Laboratory (institution)**	**Method**	**Assay kit/platform (manufacturer)**	**LLOQ**	**ULOQ**	**Imputed value if <LLOQ[a]**	**Imputated value if >ULOQ[a]**	**Samples <LLOQ *n* (%)**	**Samples >ULOQ *n* (%)**	**Inter-assay CV**	**Intra-assay CV**
Intact PTH	Plasma	AFBM (SickKids)	ELISA	Kit #60-3100 (Immutopics, California)	1.58 pmol/L	–	0.79	NR	Baseline: 32 (6.0) Delivery: 86 (16)	N/A	16%	4.2%
Whole PTH	Plasma	AFBM (SickKids)	ELISA	Kit #60-3000 (Immutopics, California)	1.58 pmol/L	–	0.79	NR	Baseline: 57 (12) Delivery: 94 (19)	N/A	17%	2.4%
25(OH)D[b]	Serum	AFBM (SickKids)	LC-MS/MS	Agilent 1290 HPLC system (Agilent Technologies; Q-TRAP 5500 mass spectrometer (Sciex))	1.25 nmol/L	–	NR	NR	N/A	N/A	10 %	5 %
FGF23	Plasma	AFBM (SickKids)	ELISA	Kit #60-600, (Immutopics, California)	9.1 RU/mL	1415 RU/mL	4.55 RU/mL	1415 RU/mL	Baseline: 1 (0) Delivery: 0 (0)	Baseline: 10 (1.8) Delivery: 16 (3.0)	6.0%	2.9%
Magnesium[c]	Serum	SickKids	Colorometric assay	DIMG-250 (BioAssays, California)	0.041 mmol/L	–	NR	NR	N/A	N/A	3.0%	7.6%
CRP[d]	Plasma	AFBM (SickKids)	ELISA	DCRP00 (R&D Systems, Minnesota)	0.78 mg/L	50 mg/L	0.39 mg/L	Extrapolated using standard curve	Baseline: 10 (7.1) Delivery: 17 (3.2)	Baseline: 1 (0.7) Delivery: 45 (8.4)	10%	5.1%

PTH, parathyroid hormone; AFBM, Analytical Facility for Bioactive Molecules; ELISA, Enzyme-linked immunosorbent assay; 25(OH)D, 25-hydroxyvitamin D; LC-MS/MS, liquid chromatography-tandem mass spectrometry; NR, not required; N/A, not applicable; SickKids, Hospital for Sick Children, Toronto, Canada.

[a]NR denoted for biomarkers for which all measured concentrations were within the quantification limits.

[b]The AFBM lab participates in the Vitamin D External Quality Assessment Scheme (DEQAS). Only 25(OH)D_3 concentrations were used in analyses because 25(OH)D_2 was always undetectable in this cohort.

[c]Samples went through 1 freeze-thaw cycle before analysis.

[d]Samples diluted 1000-fold.
