# Supplementary material for: Higher maternal parathyroid hormone concentration at delivery is not associated with smaller newborn size
Source: Endocr Connect. 2021 Feb 23;10(3):345–57. doi: 10.1530/EC-21-0056 (PMC8052570; doi:10.1530/EC-21-0056)
Supplement: Supplementary Table 2. Maternal and infant demographic and clinical information of participants in the MDIG trial cohort who were included in this study, compared to those participants who were not included. [file supplementary_table_2.pdf]

Supplementary Table 2. Maternal and infant demographic and clinical information of participants in the MDIG trial cohort who were included in this study, compared to those participants who were not included.

**Enrolled participants, N**	**Included Participants 537**	**Excluded Participants 761**	**p[a]**
**Maternal Baseline Characteristics**			
Age (years), median (min, max)	23 (18, 38)	22 (18, 40)	0.19
Marital status, n (%)[b]			0.17
Married	535 (99)	746 (100)	
Not Married	2 (0.37)	0 (0)	
Level of education, n (%)			0.06
Little to no schooling	193 (36)	321 (42)	
Some or completed secondary education	283 (53)	371 (49)	
Some or completed tertiary education	61 (11)	69 (9.1)	
Primary occupation, n (%)[b]			0.021
Homemaker	513 (96)	689 (92)	
Other	24 (4.5)	57 (7.6)	
Asset index quintiles, n (%)[c]			0.79
1 (lowest)	109 (20)	152 (20)	
2	103 (19)	148 (20)	
3	100 (19)	156 (21)	
4	114 (21)	143 (19)	
5 (highest)	110 (21)	145 (19)	
Gravidity, median (min, max)	2 (1, 9)	2 (1, 7)	0.69
Height (cm), mean ± SD	151.0 ± 5.5	150.9 ± 5.3	0.89
Estimated dietary calcium intake (mg/day), mean ± SD	984.0 ± 272.6	956.5 ± 287.1	0.08
Estimated dietary protein intake (g/kg/day), mean ± SD	0.9 ± 0.4	0.9 ± 0.4	0.21
**Delivery Characteristics**			
Gestational age at birth (weeks)[d]			
Median (minimum, maximum)	39.1 (33, 43)	39.1 (26, 43)	0.08
Preterm (<37 weeks), n (%)	33 (6.1)	91 (13)	<0.001
Term (≥37 weeks), n (%)	504 (94)	626 (87)	
Mode of delivery, n (%)[d]			<0.001
Vaginal birth	206 (38)	394 (55)	
Caesarean section	331 (62)	323 (45)	
Infant sex, n (%)[d]			0.92
Boys	272 (51)	361 (50)	
Girls	265 (49)	356 (50)	
Season of birth, n (%)[d]			0.013
Spring (March-May)	85 (16)	98 (14)	
Summer (June-August)	129 (24)	231 (32)	
Fall (September-November)	180 (34)	227 (32)	
Winter (December-February)	143 (27)	161 (22)	
Infant anthropometry, mean ± SD			
Birthweight (g)[e]	2728.6 ± 349.2	2690.8 ± 372.6	0.14
Length at birth (cm)[f]	47.5 ± 1.9	47.3 ± 2.1	0.30
Head circumference at birth (cm)[g]	33.0 ± 1.2	32.9 ± 1.2	0.28
Rump-to-knee length (cm)[h]	13.4 ± 0.8	13.3 ± 0.7	0.31
Gestational age/sex-standardized growth parameter, mean ± SD			
WAZ at birth[e]	-1.17 ± 0.85	-1.23 ± 0.89	0.34
LAZ at birth[f]	-0.89 ± 0.99	-0.94 ± 1.04	0.50
HCAZ at birth[g]	-0.60 ± 0.95	-0.66 ± 0.98	0.45
SGA, n (%)[e]	247 (46)	150 (50)	0.29

[a] P-values presented are from Chi-square or Fischer Exact Tests for categorical values and from ANOVA or Kruskal-Wallis test for continuous variables.
[b] $N_{Excluded}$=746
[c] $N_{Included}$=744; $N_{Excluded}$=536
[d] $N_{Excluded}$=717
[e] $N_{Included}$=535; $N_{Excluded}$=300
[f] $N_{Included}$=530; $N_{Excluded}$=294
[g] $N_{Included}$=534; $N_{Excluded}$=296
[h] $N_{Included}$ = 532; $N_{Excluded}$= 299
