# Supplementary material for: Higher maternal parathyroid hormone concentration at delivery is not associated with smaller newborn size
Source: Endocr Connect. 2021 Feb 23;10(3):345–57. doi: 10.1530/EC-21-0056 (PMC8052570; doi:10.1530/EC-21-0056)
Supplement: Supplementary Table 3. Baseline and delivery iPTH and wPTH concentrations, stratified by vitamin D status and calcium intake among women randomized to the placebo group. [file supplementary_table_3.pdf]

Supplementary Table 3. Baseline and delivery iPTH and wPTH concentrations, stratified by vitamin D status and calcium intake among women randomized to the placebo group.

	N_{iPTH}	iPTH, pmol/L[a]	N_{wPTH}	wPTH, pmol/L[a]
**Baseline (17-24 weeks' gestation)**	141	3.40 (2.35, 5.92)	97	2.65 (1.82, 4.07)
25(OH)D, nmol/L[b]				
<30	87	4.00 (2.39, 6.76)	62	2.71 (1.79, 4.46)
30–50	47	3.02 (2.22, 4.83)	31	2.88 (2.15, 3.47)
≥50	7	2.55 (1.69, 5.22)	4	1.66 (0.79, 6.20)
Calcium Intake Percentile[c]				
<50th	66	3.84 (2.54, 6.21)	46	2.89 (1.88, 4.00)
≥50th	75	3.04 (2.09, 5.22)	51	2.53 (1.81, 4.23)
25(OH)D/Calcium Intake				
<30/<50th	41	4.50 (2.79, 6.83)	27	3.58 (1.82, 4.66)
<30/≥50th	46	3.23 (1.84, 5.84)	35	2.34 (0.79, 4.38)
≥30/<50th	25	3.01 (2.14, 4.49)	19	2.54 (1.88, 3.25)
≥30/≥50th	29	2.98 (2.22, 5.12)	16	2.89 (2.23, 4.15)
**Delivery**	105	4.97 (3.40, 7.30)	96	4.17 (2.69, 6.07)
25(OH)D, nmol/L[d]				
<30	77	5.16 (3.54, 7.83)	69	4.55 (3.02, 6.45)
30–50	23	3.59 (1.70, 5.33)	23	2.86 (2.45, 3.74)
≥50	5	3.64 (3.43, 4.47)	4	2.94 (2.29, 4.71)
Calcium Intake Percentile[c]				
<50th	49	4.87 (3.43, 6.77)	45	3.72 (2.66, 5.95)
≥50th	56	4.98 (3.39, 7.76)	51	4.25 (2.72, 6.17)
25(OH)D/Calcium Intake Percentile				
<30/<50th	36	5.24 (3.78, 7.39)	32	4.79 (2.96, 6.51)
<30/≥50th	41	5.14 (3.54, 8.07)	37	4.35 (3.13, 6.17)
≥30/<50th	13	3.71 (3.27, 4.47)	13	2.86 (2.61, 3.31)
≥30/≥50th	15	3.59 (1.70, 6.01)	14	2.98 (1.84, 4.79)

[a] Data is presented as median concentration (25th percentile, 75th percentile).
[b] 25(OH)D concentrations measured at enrollment (17-24 weeks gestation).
[c] Calcium intake was collected from a food frequency questionnaire administered at baseline; the food frequency questionnaire was not administered again at delivery.
[d] 25(OH)D concentrations measured within -19 to 4 days of delivery (median: 0 days).
