# Supplementary material for: Higher maternal parathyroid hormone concentration at delivery is not associated with smaller newborn size
Source: Endocr Connect. 2021 Feb 23;10(3):345–57. doi: 10.1530/EC-21-0056 (PMC8052570; doi:10.1530/EC-21-0056)
Supplement: Supplementary Table 4. Multivariable linear regression analysis of the association of maternal delivery iPTH concentrations with infant length-for-age Z scores (LAZ) at birth. [file supplementary_table_4.pdf]

Supplementary Table 4. Multivariable linear regression analysis of the association of maternal delivery iPTH concentrations with infant length-for-age Z scores (LAZ) at birth.

	Unadjusted Models[a]				***Multivariable Model***[b]			
	N	Difference in LAZ	95% CI	*P*[c]	N	Difference in LAZ	95% CI	*P*[c]
Maternal log iPTH[d]	530	0.091	0.020, 0.161	0.012	487	0.064	-0.011, 0.139	0.09
Maternal Magnesium (mmol/L)	489	-0.379	-0.966, 0.209	0.21	487	-0.479	-1.026, 0.068	0.09
Maternal log FGF23[e]	530	0.022	0.013, 0.031	<0.001	487	0.019	0.011, 0.028	<0.001
Maternal log CRP[e]	529	0.003	-0.003, 0.010	0.33	487	0.004	-0.002, 0.010	0.24
Vitamin D Treatment Group								
Placebo	140	ref	ref	ref	102	ref	ref	ref
4200 IU/week	95	-0.222	-0.482, 0.037	0.09	93	-0.215	-0.478, 0.048	0.11
16800 IU/week	108	-0.086	-0.336, 0.164	0.50	107	-0.123	-0.384, 0.138	0.36
28000 IU/week	187	-0.194	-0.412, 0.024	0.08	185	-0.115	-0.351, 0.121	0.34
Estimated Protein Intake (g/kg/day)	530	-0.537	-0.759, -0.759	<0.001	487	-0.325	-0.558, -0.558	0.007
Maternal Age (years)	530	0.020	0.001, 0.040	0.044	487	0.003	-0.023, 0.029	0.82
Maternal Height (cm)	530	0.056	0.042, 0.071	<0.001	487	0.050	0.034, 0.065	<0.001
Maternal Education								
Little to no schooling	188	ref	ref	ref	176	ref	ref	ref
Some or completed secondary education	281	0.115	-0.068, 0.299	0.22	253	0.077	-0.110, 0.263	0.42
Some or completed tertiary education	61	-0.140	-0.428, 0.147	0.34	58	-0.015	-0.312, 0.282	0.92
Asset Index[f]	529	0.036	-0.014, 0.087	0.16	487	0.009	-0.014, 0.062	0.73
Gravidity	530	0.086	0.011, 0.162	0.025	487	0.050	-0.054, 0.153	0.35
Gestational age at birth (weeks)	530	-0.079	-0.140, -0.018	0.012	487	-0.047	-0.109, 0.014	0.13
Season of Birth[g]								
Spring	85	ref	ref	ref	79	ref	ref	ref
Summer	127	-0.182	-0.454, 0.090	0.19	118	-0.202	-0.465, 0.061	0.13
Autumn	178	0.010	-0.246, 0.267	0.94	162	-0.093	-0.341, 0.155	0.46
Winter	140	-0.299	-0.566, -0.032	0.028	128	-0.295	-0.552, -0.038	0.025

[a] Separate univariate models were run for each listed covariate.

[b] Multivariable model adjusted for: maternal log iPTH, maternal magnesium concentrations (mmol/L), maternal log FGF23 concentrations, maternal log CRP concentrations, vitamin D supplementation group (Placebo, 4200 IU/week, 16800 IU/week, 28000 IU/week), estimated protein intake (g/kg/day), maternal age (years), maternal height (cm), maternal education (little to no schooling, some or completed secondary education, some or completed tertiary education), gravidity, gestational age at birth (weeks), season of birth (spring, summer, fall, winter).

[c] $p<0.05$ considered significant.

[d] Variable was log transformed; Regression coefficient represents estimated mean difference in LAZ for a 90% increase in iPTH concentrations, which reflects a large but plausible difference in iPTH concentration that corresponds to the observed effect on iPTH of high-dose vitamin D (28,000 IU/week), versus placebo.

[e] Variable was log transformed; Regression coefficient represents estimated mean difference in LAZ per 10% increase in biomarker concentrations.

[f] Derived by data reduction using principal component analysis as measure of indicators of socioeconomic status including: private toilet, electricity, radio, television, mobile phone, landline, fridge, Almirah (wardrobe), table, chair(s), electric fan, DVD player, auto-bike, rickshaw/van, bicycle, motorcycle/motor scooter/ temp/CNG, livestock/herds/farm animals/poultry, homestead, and land. The first principal component was used to assign each individual an asset score; lower scores reflect lower relative wealth and higher scores indicate greater wealth.

[g] Spring: March-May; Summer: June-August; Autumn: September-November; Winter: December-February.
