# Supplementary material for: Higher maternal parathyroid hormone concentration at delivery is not associated with smaller newborn size
Source: Endocr Connect. 2021 Feb 23;10(3):345–57. doi: 10.1530/EC-21-0056 (PMC8052570; doi:10.1530/EC-21-0056)
Supplement: Supplementary Table 5. Multivariable linear regression analysis of the association of maternal delivery iPTH concentrations with infant weight-for-age Z-scores (WAZ) at birth. [file supplementary_table_5.pdf]

Supplementary Table 5. Multivariable linear regression analysis of the association of maternal delivery iPTH concentrations with infant weight-for-age Z-scores (WAZ) at birth.

	Unadjusted Models[a]				*Multivariable Model*[b]			
	N	Difference in WAZ	95% CI	*P*[c]	N	Difference in WAZ	95% CI	*P*[c]
Maternal log iPTH[d]	535	0.070	0.010, 0.129	0.022	490	0.056	-0.007, 0.118	0.08
Maternal Magnesium (mmol/L)	493	-0.241	-0.743, 0.261	0.35	490	-0.283	-0.745, 0.179	0.23
Maternal log FGF23[e]	535	0.020	0.013, 0.028	<0.001	490	0.016	0.009, 0.024	<0.001
Maternal log CRP[e]	533	-0.001	-0.007, 0.004	0.66	490	0.000	-0.005, 0.005	0.93
Vitamin D Treatment Group								
Placebo	141	ref	ref	ref	102	ref	ref	ref
4200 IU/week	96	-0.143	-0.363, 0.078	0.20	94	-0.173	-0.393, 0.047	0.12
16800 IU/week	108	0.038	-0.175, 0.251	0.73	106	-0.058	-0.279, 0.162	0.60
28000 IU/week	190	-0.079	-0.264, 0.106	0.40	188	-0.033	-0.231, 0.165	0.74
Estimated Protein Intake (g/kg/day)	535	-0.563	-0.754, -0.754	<0.001	490	-0.393	-0.593, -0.593	<0.001
Maternal Age (years)	535	0.029	0.012, 0.046	0.001	490	0.004	-0.018, 0.026	0.73
Maternal Height (cm)	535	0.031	0.018, 0.043	<0.001	490	0.023	0.010, 0.035	0.001
Maternal Education								
Little to no schooling	193	ref	ref	ref	180	ref	ref	ref
Some or completed secondary education	281	0.074	-0.081, 0.229	0.35	252	0.061	-0.095, 0.217	0.44
Some or completed tertiary education	61	-0.173	-0.417, 0.072	0.17	58	-0.050	-0.299, 0.199	0.69
Asset Index[f]	534	0.038	-0.005, 0.081	0.08	490	0.032	-0.005, 0.076	0.15
Gravidity	535	0.136	0.073, 0.199	<0.001	490	0.070	-0.016, 0.157	0.11
Gestational age at birth (weeks)	535	-0.175	-0.224, -0.125	<0.001	490	-0.144	-0.196, -0.093	<0.001
Season of Birth[g]								
Spring	85	ref	ref	ref	79	ref	ref	ref
Summer	128	-0.073	-0.305, 0.159	0.54	118	-0.126	-0.347, 0.094	0.26
Autumn	179	0.017	-0.201, 0.236	0.88	163	-0.050	-0.257, 0.158	0.64
Winter	143	-0.223	-0.451, 0.004	0.05	130	-0.171	-0.387, 0.044	0.12

[a] Separate univariate models were run for each listed covariate.
[b] Multivariable model adjusted for: maternal log iPTH, maternal magnesium concentrations (mmol/L), maternal log FGF23 concentrations, maternal log CRP concentrations, vitamin D supplementation group (Placebo, 4200 IU/week, 16800 IU/week, 28000 IU/week), estimated protein intake (g/kg/day), maternal age (years), maternal height (cm), maternal education (little to no schooling, some or completed secondary education, some or completed tertiary education), gravidity, gestational age at birth (weeks), season of birth (spring, summer, fall, winter).
[c] p<0.05 considered significant.
[d] Variable was log transformed; Regression coefficient represents estimated mean difference in WAZ for a 90% increase in iPTH concentrations, which reflects a large but plausible difference in iPTH concentration that corresponds to the observed effect of high-dose vitamin D (28,000 IU/week) on iPTH, versus placebo.
[e] Variable was log transformed; Regression coefficient represents estimated mean difference in WAZ for a 10% increase in biomarker concentrations.
[f] Derived by data reduction using principal component analysis as measure of indicators of socioeconomic status including: private toilet, electricity, radio, television, mobile phone, landline, fridge, Almirah (wardrobe), table, chair(s), electric fan, DVD player, auto-bike, rickshaw/van, bicycle, motorcycle/motor scooter/ temp/CNG, livestock/herds/ farm animals/poultry, homestead, and land. The first principal component was used to assign each individual an asset score; lower scores reflect lower relative wealth and higher scores indicate greater wealth.
[g] Spring: March-May; Summer: June-August; Autumn: September-November; Winter: December-February.
