# Supplementary material for: Higher maternal parathyroid hormone concentration at delivery is not associated with smaller newborn size
Source: Endocr Connect. 2021 Feb 23;10(3):345–57. doi: 10.1530/EC-21-0056 (PMC8052570; doi:10.1530/EC-21-0056)
Supplement: Supplementary Table 7. Multivariable linear regression analysis of the association of maternal delivery iPTH concentrations with rump-to-knee length (RKL). [file supplementary_table_7.pdf]

Supplementary Table 7. Multivariable linear regression analysis of the association of maternal delivery iPTH concentrations with rump-to-knee length (RKL).

	Unadjusted Models[a]				*Multivariable Model*[b]			
	N	Difference in RKL (cm)	95% CI	*P*[c]	N	Difference in RKL (cm)	95% CI	*P*[c]
Maternal log iPTH[d]	532	0.004	-0.050, 0.058	0.89	487	-0.004	-0.062, 0.053	0.88
Maternal Magnesium (mmol/L)	490	0.174	-0.271, 0.620	0.44	487	0.223	-0.197, 0.642	0.30
Maternal log FGF23[e]	532	0.010	0.003, 0.017	0.006	487	0.010	0.003, 0.017	0.004
Maternal log CRP[e]	530	0.003	-0.002, 0.007	0.31	487	0.003	-0.002, 0.007	0.27
Vitamin D Treatment Group								
Placebo	142	ref	ref	ref	103	ref	ref	ref
4200 IU/week	95	0.035	-0.162, 0.233	0.73	93	-0.090	-0.292, 0.111	0.38
16800 IU/week	108	0.120	-0.070, 0.310	0.22	106	0.098	-0.102, 0.298	0.34
28000 IU/week	187	0.125	-0.041, 0.291	0.14	185	0.024	-0.157, 0.206	0.79
Estimated Protein Intake (g/kg/day)	532	-0.129	-0.302, 0.044	0.14	487	-0.108	-0.287, 0.072	0.24
Maternal Age (years)	532	-0.001	-0.016, 0.014	0.90	487	0.007	-0.013, 0.027	0.47
Maternal Height (cm)	532	0.029	0.017, 0.040	<0.001	487	0.028	0.016, 0.039	<0.001
Maternal Education								
Little to no schooling	192	ref	ref	ref	179	ref	ref	ref
Some or completed secondary education	279	-0.026	-0.166, 0.114	0.72	250	-0.051	-0.194, 0.092	0.49
Some or completed tertiary education	61	0.051	-0.168, 0.270	0.65	58	-0.017	-0.245, 0.210	0.88
Asset Index[f]	531	0.037	-0.001, 0.076	0.06	487	0.007	-0.001, 0.047	0.73
Gravidity	532	-0.013	-0.071, 0.045	0.65	487	-0.014	-0.094, 0.065	0.72
Gestational age at birth (weeks)	532	0.133	0.088, 0.178	<0.001	487	0.126	0.078, 0.173	<0.001
Infant Sex								
Male	268	ref	ref	ref	251	ref	ref	ref
Female	264	-0.072	-0.201, 0.057	0.27	236	-0.080	-0.205, 0.045	0.21
Season of Birth[g]								
Spring	85	ref	ref	ref	79	ref	ref	ref
Summer	127	-0.333	-0.540, -0.126	0.002	117	-0.264	-0.466, -0.063	0.010
Autumn	178	-0.294	-0.489, -0.100	0.003	162	-0.387	-0.577, -0.197	<0.001
Winter	142	-0.129	-0.332, 0.073	0.21	129	-0.096	-0.293, 0.102	0.34

[a] Separate univariate models were run for each listed covariate.
[b] Multivariable model adjusted for: maternal log iPTH, maternal magnesium concentrations (mmol/L), maternal log FGF23 concentrations, maternal log CRP concentrations, vitamin D supplementation group (Placebo, 4200 IU/week, 16800 IU/week, 28000 IU/week), estimated protein intake (g/kg/day), maternal age (years), maternal height (cm), maternal education (little to no schooling, some or completed secondary education, some or completed tertiary education), gravidity, gestational age at birth (weeks), season of birth (spring, summer, fall, winter), and infant sex.
[c] $p<0.05$ considered significant.
[d] Variable was log transformed; Regression coefficient represents the difference in RKL for a 90% increase in iPTH concentrations, which reflects a large but plausible difference in iPTH concentration that corresponds to the observed effect of high-dose vitamin D (28,000 IU/week) on iPTH, versus placebo.
[e] Variable was log transformed; Regression coefficient represents estimated difference in RKL for a 10% increase in biomarker concentrations.
[f] Derived by data reduction using principal component analysis as measure of indicators of socioeconomic status including: private toilet, electricity, radio, television, mobile phone, landline, fridge, Almirah (wardrobe), table, chair(s), electric fan, DVD player, auto-bike, rickshaw/van, bicycle, motorcycle/motor scooter/ temp/CNG, livestock/herds/ farm animals/poultry, homestead, and land. The first principal component was used to assign each individual an asset score; lower scores reflect lower relative wealth and higher scores indicate greater wealth.
[g] Spring: March-May; Summer: June-August; Autumn: September-November; Winter: December-February.
