# Supplementary material for: Higher maternal parathyroid hormone concentration at delivery is not associated with smaller newborn size
Source: Endocr Connect. 2021 Feb 23;10(3):345–57. doi: 10.1530/EC-21-0056 (PMC8052570; doi:10.1530/EC-21-0056)
Supplement: Supplementary Table 8. Multivariable modified poisson regression with robust error variance of the association of maternal delivery iPTH concentrations with risk of small-for-gestational age (SGA) [file supplementary_table_8.pdf]

Supplementary Table 8. Multivariable modified poisson regression with robust error variance of the association of maternal delivery iPTH concentrations with risk of small-for-gestational age (SGA).

	Unadjusted Models[a]				***Multivariable Model***[b]			
	N	Relative Risk	95% CI	*P*[c]	N	Relative Risk	95% CI	*P*[c]
Maternal log iPTH[d]	535	0.92	0.85, 0.99	0.023	490	0.94	0.87, 1.02	0.17
Maternal Magnesium (mmol/L)	493	1.20	0.67, 2.15	0.54	490	1.36	0.81, 2.29	0.25
Maternal log FGF23[e]	535	0.98	0.97, 0.99	<0.001	490	0.98	0.97, 0.99	0.003
Maternal log CRP[e]	533	1.01	1.00, 1.01	0.047	490	1.01	1.00, 1.01	0.035
Vitamin D Treatment Group								
Placebo	141	ref	ref	ref	102	ref	ref	ref
4200 IU/week	96	1.15	0.86, 1.52	0.35	94	1.22	0.90, 1.65	0.20
16800 IU/week	108	1.04	0.78, 1.39	0.79	106	1.21	0.87, 1.67	0.25
28000 IU/week	190	1.19	0.94, 1.52	0.15	188	1.17	0.88, 1.57	0.28
Estimated Protein Intake (g/kg/day)	535	1.81	1.51, 1.91	<0.001	490	1.55	1.24, 1.93	<0.001
Maternal Age (years)	535	0.97	0.95, 0.99	0.006	490	0.99	0.96, 1.02	0.44
Maternal Height (cm)	535	0.97	0.95, 0.99	<0.001	490	0.98	0.97, 1.00	0.047
Maternal Education								
Little to no schooling	193	ref	ref	ref	180	ref	ref	ref
Some or completed secondary education	281	0.92	0.75, 1.13	0.44	252	0.90	0.72, 1.12	0.33
Some or completed tertiary education	61	1.27	0.98, 1.64	0.07	58	1.10	0.82, 1.48	0.52
Asset Index[f]	534	0.98	0.93, 1.04	0.46	490	0.97	0.93, 1.04	0.38
Gravidity	535	0.88	0.80, 0.97	0.011	490	0.97	0.86, 1.09	0.63
Gestational age at birth (weeks)	535	1.21	1.14, 1.29	<0.001	490	1.17	1.10, 1.26	<0.001
Season of Birth[g]								
Spring	85	ref	ref	ref	79	ref	ref	ref
Summer	128	1.19	0.87, 1.64	0.28	118	1.30	0.95, 1.77	0.10
Autumn	179	0.99	0.72, 1.36	0.96	163	1.10	0.81, 1.49	0.55
Winter	143	1.42	1.05, 1.91	0.022	130	1.36	1.02, 1.82	0.039

[a] Separate univariate models were run for each listed covariate.
[b] Multivariable model adjusted for: maternal log iPTH, maternal magnesium concentrations (mmol/L), maternal log FGF23 concentrations, maternal log CRP concentrations, vitamin D supplementation group (Placebo, 4200 IU/week, 16800 IU/week, 28000 IU/week), estimated protein intake (g/kg/day), maternal age (years), maternal height (cm), maternal education (little to no schooling, some or completed secondary education, some or completed tertiary education), gravidity, gestational age at birth (weeks), season of birth (spring, summer, fall, winter).
[c] $p<0.05$ considered significant.
[d] Variable was log transformed; Regression coefficient represents the relative risk of SGA for a 90% increase in iPTH concentrations, which reflects a large but plausible difference in iPTH concentration that corresponds to the observed effect of high-dose vitamin D (28,000 IU/week) on iPTH, versus placebo.
[e] Variable was log transformed; Regression coefficient represents estimated relative risk of SGA for a 10% increase in biomarker concentrations.
[f] Derived by data reduction using principal component analysis as measure of indicators of socioeconomic status including: private toilet, electricity, radio, television, mobile phone, landline, fridge, Almirah (wardrobe), table, chair(s), electric fan, DVD player, auto-bike, rickshaw/van, bicycle, motorcycle/motor scooter/ temp/CNG, livestock/herds/ farm animals/poultry, homestead, and land. The first principal component was used to assign each individual an asset score; lower scores reflect lower relative wealth and higher scores indicate greater wealth.
[g] Spring: March-May; Summer: June-August; Autumn: September-November; Winter: December-February.
