# Supplementary material for: Higher maternal parathyroid hormone concentration at delivery is not associated with smaller newborn size
Source: Endocr Connect. 2021 Feb 23;10(3):345–57. doi: 10.1530/EC-21-0056 (PMC8052570; doi:10.1530/EC-21-0056)
Supplement: Supplementary Table 9. Multivariable linear regression analysis of the association of maternal iPTH or wPTH concentrations at enrollment (17-24 weeks of gestation) among women randomized to the placebo group with infant LAZ, WAZ, HCAZ, and RKL at birtha. [file supplementary_table_9.pdf]

Supplementary Table 9. Multivariable linear regression analysis of the association of maternal iPTH or wPTH concentrations at enrollment (17-24 weeks of gestation) among women randomized to the placebo group with infant LAZ, WAZ, HCAZ, and RKL at birth[a].

	Unadjusted Models				*Multivariable Models* [b]			
	N	Effect Size[c]	95% CI	P^d	N	Effect Size[c]	95% CI	P^d
**LAZ**								
log iPTH[e]	140	0.093	-0.054, 0.240	0.21	138	0.067	-0.082, 0.216	0.37
log wPTH[f]	96	0.083	-0.078, 0.243	0.31	95	0.042	-0.124, 0.208	0.62
**WAZ**								
log iPTH[e]	141	0.050	-0.070, 0.169	0.41	139	0.010	-0.103, 0.123	0.86
log wPTH[f]	96	0.074	-0.053, 0.202	0.25	95	0.011	-0.116, 0.137	0.87
**HCAZ**								
log iPTH[e]	142	0.035	-0.104, 0.175	0.61	140	-0.012	-0.157, 0.134	0.88
log wPTH[f]	97	0.046	-0.093, 0.185	0.51	96	-0.014	-0.153, 0.126	0.85
**RKL**								
log iPTH[e]	142	-0.025	-0.133, 0.084	0.66	140	-0.007	-0.117, 0.103	0.90
log wPTH[f]	97	-0.012	-0.128, 0.105	0.85	96	-0.032	-0.148, 0.084	0.58
**SGA**								
log iPTH[e]	141	0.94	0.71, 1.26	0.69	139	1.00	0.84, 1.19	0.98
log wPTH[f]	96	0.88	0.64, 1.21	0.43	95	0.94	0.74, 1.18	0.59

[a] LAZ, length-for-gestational age z-scores; WAZ, weight-for-gestational age z-scores; HCAZ, head circumference-for-gestational age z-scores; SGA, small-for-gestational age.
[b] Multivariable model adjusted for: maternal log wPTH or iPTH, maternal baseline log FGF23 concentrations, maternal baseline log CRP concentrations, 25(OH)D concentrations at baseline, maternal age (years), maternal height (cm), maternal education (no schooling, some or completed primary education, some or completed secondary education, some or completed tertiary education), gravidity, gestational age at birth (weeks), season of birth (spring, summer, fall, winter). The model where RKL was the outcome additionally adjusted for sex of the infant.
[c] For models where LAZ, WAZ, HCAZ, or RKL is the outcome, effect estimate represents difference in anthropometric outcome (obtained from linear regression models). For models where SGA is the outcome, effect estimates represent the relative risk of SGA (obtained from a Poisson regression model with robust error variance).
[d] $p<0.05$ considered significant.
[e] Variable was log transformed; regression coefficient represents estimated mean difference in LAZ per 90% increase in iPTH concentrations.
[f] Variable was log transformed; Regression coefficient represents estimated mean difference in LAZ per 73% increase in wPTH concentrations.
