# Supplementary material for: Higher maternal parathyroid hormone concentration at delivery is not associated with smaller newborn size
Source: Endocr Connect. 2021 Feb 23;10(3):345–57. doi: 10.1530/EC-21-0056 (PMC8052570; doi:10.1530/EC-21-0056)
Supplement: Supplementary Table 10. Multivariable linear regression analysis of the association of maternal calcium and protein intake, magnesium concentrations, fibroblast like growth factor 23 (FGF23), C-reactive protein (CRP), 25-hydroxyvitamin D (25(OH)D) and vitamin D supplementation with maternal delivery [file supplementary_table_10.pdf]

Supplementary Table 10. Multivariable linear regression analysis of the association of maternal calcium and protein intake, magnesium concentrations, fibroblast like growth factor 23 (FGF23), C-reactive protein (CRP), 25-hydroxyvitamin D (25(OH)D) and vitamin D supplementation with maternal delivery log intact PTH (iPTH) concentrations

	Unadjusted Models				***Multivariable Model***[b]			
	N	Difference in log iPTH	95% CI	P^a	N	Difference in log iPTH	95% CI	P^a
Estimated Calcium Intake (mg/day)	537	0.003	-0.021, 0.027	0.82	492	-0.002	-0.038, 0.034	0.92
Maternal Magnesium (mmol/L)	495	0.460	0.015, 0.904	0.043	492	0.255	-0.170, 0.680	0.24
Maternal log FGF23[c]	537	0.090	0.016, 0.164	0.017	492	0.084	0.012, 0.156	0.022
Maternal log CRP[c]	535	-0.014	-0.065, 0.038	0.61	492	-0.026	-0.076, 0.024	0.31
Vitamin D Treatment Group								
Placebo	142	ref	ref	ref	103	ref	ref	ref
2,400 IU/week	96	-0.383	-0.569, -0.198	<0.001	94	-0.281	-0.483, -0.079	0.006
16,800 IU/week	109	-0.701	-0.879, -0.522	<0.001	107	-0.632	-0.826, -0.438	<0.001
28,000 IU/week	190	-0.710	-0.865, -0.554	<0.001	188	-0.599	-0.773, -0.425	<0.001
Maternal log 25(OH)D[c, d]	497	-0.357	-0.440, -0.275	<0.001	490	-0.346	-0.431, -0.262	<0.001
Estimated Protein Intake (g/kg/day)	537	-0.008	-0.184, 0.168	0.93	492	-0.011	-0.282, 0.261	0.94
Maternal Age (years)	537	-0.005	-0.021, 0.010	0.49	492	-0.013	-0.034, 0.007	0.20
Maternal Education								
Little to no schooling	193	ref	ref	ref	180	ref	ref	ref
Some or completed secondary education	283	0.114	-0.027, 0.255	0.11	254	0.078	-0.066, 0.222	0.29
Some or completed tertiary education	61	-0.111	-0.333, 0.111	0.33	58	-0.003	-0.234, 0.227	0.98
Asset Index[e]	536	0.019	-0.020, 0.058	0.35	492	0.023	-0.020, 0.063	0.27
Gravidity	537	0.017	-0.041, 0.076	0.56	492	0.036	-0.044, 0.116	0.38

[a] $p<0.05$ considered significant.
[b] Multivariable model adjusted for: daily calcium intake, maternal magnesium, maternal log FGF23, maternal log CRP, vitamin D treatment group, maternal age, maternal education, asset index, and gravidity.
[c] Variable was log transformed; Regression coefficient represents the difference in log iPTH concentrations for every unit increase in the log-transformed predictor variable.
[d] In a separate multivariable model, the association of maternal log 25(OH)D with PTH concentrations was estimated, substituting vitamin D treatment group with 25(OH)D. In addition to 25(OH)D, this model included: Daily calcium intake, maternal magnesium, maternal log FGF23, maternal log CRP, maternal age, maternal education, asset index, and gravidity.
[e] Derived by data reduction using principal component analysis as measure of indicators of socioeconomic status. SES indicators measured in a baseline survey data from MDIG trial include: private toilet, electricity, radio, television, mobile phone, landline, fridge, almirah (wardrobe), table, chair(s), electric fan, DVD player, auto-bike, rickshaw/van, bicycle, motorcycle/motor scooter/temp/CNG, livestock/herds/farm animals/poultry, homestead, and land. The first principal component was used to assign each individual an asset score; lower scores reflect ownership of fewer items (lower relative wealth), and higher scores indicate greater wealth.
