# Supplementary material for: Higher maternal parathyroid hormone concentration at delivery is not associated with smaller newborn size
Source: Endocr Connect. 2021 Feb 23;10(3):345–57. doi: 10.1530/EC-21-0056 (PMC8052570; doi:10.1530/EC-21-0056)
Supplement: Supplementary Table 11. Multivariable linear regression analysis of the association of maternal calcium and protein intake, magnesium concentrations, fibroblast like growth factor 23 (FGF23), C-reactive protein (CRP), 25-hydroxyvitamin D (25(OH)D) and vitamin D supplementation with maternal delivery [file supplementary_table_11.pdf]

Supplementary Table 11. Multivariable linear regression analysis of the association of maternal calcium and protein intake, magnesium concentrations, fibroblast like growth factor 23 (FGF23), C-reactive protein (CRP), 25-hydroxyvitamin D (25(OH)D) and vitamin D supplementation with maternal delivery log whole PTH (wPTH) concentrations.

	Unadjusted Models				***Multivariable Model***[b]			
	N	Difference in log wPTH	95% CI	*P*[a]	N	Difference in log wPTH	95% CI	*P*[a]
Estimated Calcium Intake (mg/day)	492	0.000	-0.025, 0.024	0.97	484	-0.007	-0.043, 0.029	0.70
Maternal Magnesium (mmol/L)	487	0.715	0.274, 1.156	0.002	484	0.506	0.080, 0.932	0.020
Maternal log FGF23[c]	492	0.139	0.066, 0.211	<0.001	484	0.135	0.064, 0.206	<0.001
Maternal log CRP[c]	490	-0.035	-0.087, 0.017	0.19	484	-0.022	-0.072, 0.027	0.37
Vitamin D Treatment Group								
Placebo	97	ref	ref	ref	95	ref	ref	ref
2,400 IU/week	96	-0.234	-0.434, -0.033	0.022	94	-0.202	-0.404, 0.000	0.05
16,800 IU/week	109	-0.470	-0.664, -0.275	<0.001	107	-0.491	-0.685, -0.296	<0.001
28,000 IU/week	190	-0.568	-0.741, -0.394	<0.001	188	-0.517	-0.693, -0.341	<0.001
Maternal log 25(OH)D[c, d]	488	-0.294	-0.381, -0.208	<0.001	482	-0.291	-0.378, -0.204	<0.001
Estimated Protein Intake (g/kg/day)	492	0.009	-0.167, 0.186	0.92	484	0.074	-0.194, 0.343	0.59
Maternal Age (years)	492	-0.006	-0.021, 0.010	0.49	484	-0.009	-0.029, 0.011	0.39
Maternal Education								
Little to no schooling	183	ref	ref	ref	179	ref	ref	ref
Some or completed secondary education	251	0.101	-0.040, 0.242	0.16	248	0.119	-0.024, 0.261	0.10
Some or completed tertiary education	58	0.039	-0.180, 0.258	0.72	57	0.108	-0.121, 0.337	0.35
Asset Index[e]	491	0.016	-0.023, 0.055	0.41	484	0.010	-0.022, 0.050	0.64
Gravidity	492	0.011	-0.049, 0.072	0.72	484	0.040	-0.039, 0.119	0.32

[a] p<0.05 considered significant.
[b] Multivariable model adjusted for: daily calcium intake, maternal magnesium, maternal log FGF23, maternal log CRP, vitamin D treatment group, maternal age, maternal education, asset index, and gravidity.
[c] Variable was log transformed; Regression coefficient represents the difference in log iPTH concentrations for every unit increase in the log-transformed predictor variable.
[d] In a separate multivariable model, the association of maternal log 25(OH)D with PTH concentrations was estimated, substituting vitamin D treatment group with 25(OH)D. In addition to 25(OH)D, this model included: Daily calcium intake, maternal magnesium, maternal log FGF23, maternal log CRP, maternal age, maternal education, asset index, and gravidity.
[e] Derived by data reduction using principal component analysis as measure of indicators of socioeconomic status. SES indicators measured in a baseline survey data from MDIG trial include: private toilet, electricity, radio, television, mobile phone, landline, fridge, almirah (wardrobe), table, chair(s), electric fan, DVD player, auto-bike, rickshaw/van, bicycle, motorcycle/motor scooter/temp/CNG, livestock/herds/farm animals/poultry, homestead, and land. The first principal component was used to assign each individual an asset score; lower scores reflect ownership of fewer items (lower relative wealth), and higher scores indicate greater wealth.
