# Supplementary material for: Higher maternal parathyroid hormone concentration at delivery is not associated with smaller newborn size
Source: Endocr Connect. 2021 Feb 23;10(3):345–57. doi: 10.1530/EC-21-0056 (PMC8052570; doi:10.1530/EC-21-0056)
Supplement: Supplementary Table 12. Standardized direct and indirect effects of the predictors of intact PTH (iPTH), birth length-for-age z-score (LAZ), birth weight-for-age z-score (WAZ), and birth head circumference-for-age z-score (HCAZ) (n=481)a [file supplementary_table_12.pdf]

Supplementary Table 12. Standardized direct and indirect effects of the predictors of intact PTH (iPTH), birth length-for-age z-score (LAZ), birth weight-for-age z-score (WAZ), and birth head circumference-for-age z-score (HCAZ) (n=481)[a].

	Direct Effect (95% CI)	p[b]	Indirect Effect (95% CI)	p[b]	Total Effect (95% CI)	p[b]
**Maternal log iPTH**						
Maternal log 25(OH)D	-0.258 (-0.425, -0.090)	0.003	0.020 (-0.006, 0.045)	0.13	-0.238 (-0.406, -0.070)	0.006
Vitamin D Supplementation						
Placebo	ref	ref	ref	ref	ref	ref
4200 IU/week	0.022 (-0.349, 0.393)	0.91	-0.400 (-0.665, -0.134)	0.003	-0.378 (-0.646, -0.110)	0.006
16800 IU/week	-0.292 (-0.728, 0.145)	0.19	-0.532 (-0.890, -0.173)	0.004	-0.823 (-1.080, -0.566)	<0.001
28000 IU/week	-0.211 (-0.654, 0.231)	0.35	-0.598 (-0.981, -0.215)	0.002	-0.810 (-1.040, -0.580)	<0.001
Maternal log FGF23	0.107 (0.023, 0.190)	0.012	–	–	0.107 (0.023, 0.190)	0.012
Maternal log Magnesium	0.050 (-0.033, 0.133)	0.24	–	–	0.050 (-0.033, 0.133)	0.24
Estimated Protein Intake	-0.049 (-0.138, 0.039)	0.27	-0.007 (-0.022, 0.007)	0.33	-0.057 (-0.145, 0.032)	0.21
Season						
Spring	ref	ref	ref	ref	ref	ref
Summer	-0.072 (-0.337, 0.194)	0.60	-0.027 (-0.077, 0.024)	0.30	-0.098 (-0.367, 0.171)	0.48
Fall	-0.240 (-0.489, 0.009)	0.06	0.029 (-0.018, 0.077)	0.23	-0.211 (-0.463, 0.042)	0.10
Winter	-0.076 (-0.336, 0.183)	0.57	-0.002 (-0.050, 0.047)	0.95	-0.078 (-0.341, 0.185)	0.56
Maternal Age	-0.073 (-0.182, 0.037)	0.19	-0.002 (-0.022, 0.018)	0.85	-0.075 (-0.186, 0.036)	0.19
Maternal Height	-0.072 (-0.157, 0.013)	0.10	-0.006 (-0.021, 0.010)	0.48	-0.078 (-0.164, 0.008)	0.08
Gravidity	0.026 (-0.081, 0.134)	0.63	0.000 (-0.020, 0.019)	0.98	0.026 (-0.083, 0.136)	0.64
Gestational Age at Birth	-0.039 (-0.124, 0.046)	0.37	-0.016 (-0.033, 0.001)	0.07	-0.055 (-0.141, 0.031)	0.21
Asset Index	0.076 (-0.009, 0.162)	0.08	0.005 (-0.011, 0.022)	0.54	0.082 (-0.005, 0.168)	0.06
**LAZ at Birth**						
Maternal log iPTH	0.082 (0.002, 0.162)	0.045	–	–	0.082 (0.002, 0.162)	0.045
Maternal log 25(OH)D	–	–	-0.006 (-0.054, 0.043)	0.82	-0.006 (-0.054, 0.043)	0.82
Vitamin D Supplementation						
Placebo	–	–	ref	ref	ref	ref
4200 IU/week	–	–	-0.023 (-0.096, 0.050)	0.53	-0.023 (-0.096, 0.050)	0.53
16800 IU/week	–	–	-0.017 (-0.111, 0.077)	0.72	-0.017 (-0.111, 0.077)	0.72
28000 IU/week	–	–	-0.045 (-0.132, 0.043)	0.32	-0.045 (-0.132, 0.043)	0.32
Maternal log FGF23	0.196 (0.116, 0.276)	<0.001	0.009 (-0.002, 0.020)	0.12	0.204 (0.124, 0.284)	<0.001
Maternal log Magnesium	-0.086 (-0.165, -0.006)	0.034	0.004 (-0.004, 0.012)	0.31	-0.081 (-0.161, -0.002)	0.044
Estimated Protein Intake	-0.105 (-0.19, -0.021)	0.014	-0.029 (-0.054, -0.005)	0.017	-0.135 (-0.221, -0.048)	0.002
Season						
Spring	ref	ref	ref	ref	ref	ref
Summer	-0.180 (-0.433, 0.074)	0.16	-0.024 (-0.092, 0.044)	0.49	-0.204 (-0.465, 0.058)	0.13
Fall	-0.098 (-0.337, 0.141)	0.42	-0.012 (-0.076, 0.053)	0.73	-0.109 (-0.356, 0.137)	0.38
Winter	-0.294 (-0.542, -0.045)	0.02	-0.019 (-0.085, 0.047)	0.57	-0.312 (-0.569, -0.056)	0.017
Maternal Age	0.033 (-0.072, 0.138)	0.54	-0.011 (-0.039, 0.017)	0.43	0.021 (-0.087, 0.129)	0.70
Maternal Height	0.290 (0.208, 0.371)	<0.001	-0.012 (-0.034, 0.010)	0.29	0.278 (0.194, 0.362)	<0.001
Gravidity	0.039 (-0.064, 0.142)	0.46	-0.001 (-0.028, 0.026)	0.96	0.038 (-0.068, 0.145)	0.48
Gestational Age at Birth	-0.063 (-0.145, 0.018)	0.13	-0.017 (-0.039, 0.005)	0.13	-0.080 (-0.164, 0.003)	0.06
Asset Index	0.002 (-0.080, 0.084)	0.97	0.016 (-0.006, 0.039)	0.16	0.018 (-0.066, 0.102)	0.67
**WAZ at Birth**						
Maternal log iPTH	0.062 (-0.005, 0.129)	0.07	–	–	0.062 (-0.005, 0.129)	0.07
Maternal log 25(OH)D	–	–	0.000 (-0.040, 0.040)	0.99	0.000 (-0.040, 0.040)	0.99
Vitamin D Supplementation						
Placebo	–	–	ref	ref	ref	ref
4200 IU/week	–	–	-0.017 (-0.076, 0.042)	0.58	-0.017 (-0.076, 0.042)	0.58
16800 IU/week	–	–	-0.011 (-0.088, 0.067)	0.79	-0.011 (-0.088, 0.067)	0.79
28000 IU/week	–	–	-0.038 (-0.111, 0.034)	0.30	-0.038 (-0.111, 0.034)	0.30
Maternal log FGF23	0.168 (0.101, 0.235)	<0.001	0.007 (-0.002, 0.015)	0.14	0.174 (0.107, 0.241)	<0.001
Maternal log Magnesium	-0.050 (-0.116, 0.017)	0.14	0.003 (-0.003, 0.009)	0.32	-0.046 (-0.113, 0.020)	0.17
Estimated protein intake	-0.135 (-0.206, -0.064)	<0.001	-0.023 (-0.043, -0.003)	0.021	-0.158 (-0.231, -0.086)	<0.001

	Direct Effect (95% CI)	p[b]	Indirect Effect (95% CI)	p[b]	Total Effect (95% CI)	p[b]
Season						
Spring	ref	ref	ref	ref	ref	ref
Summer	-0.104 (-0.316, 0.108)	0.34	-0.023 (-0.078, 0.033)	0.42	-0.126 (-0.345, 0.092)	0.26
Fall	-0.045 (-0.245, 0.155)	0.66	-0.008 (-0.060, 0.045)	0.77	-0.053 (-0.259, 0.153)	0.62
Winter	-0.172 (-0.380, 0.036)	0.11	-0.018 (-0.071, 0.036)	0.51	-0.190 (-0.404, 0.025)	0.08
Maternal Age	0.017 (-0.071, 0.105)	0.70	-0.009 (-0.032, 0.014)	0.45	0.008 (-0.082, 0.099)	0.86
Maternal Height	0.142 (0.074, 0.210)	<0.001	-0.01 (-0.028, 0.008)	0.30	0.132 (0.062, 0.202)	<0.001
Gravidity	0.075 (-0.012, 0.161)	0.09	-0.001 (-0.023, 0.021)	0.94	0.074 (-0.015, 0.163)	0.10
Gestational Age at Birth	-0.200 (-0.268, -0.132)	<0.001	-0.014 (-0.032, 0.004)	0.13	-0.214 (-0.284, -0.144)	<0.001
Asset Index	0.047 (-0.022, 0.115)	0.18	0.014 (-0.004, 0.033)	0.13	0.061 (-0.009, 0.131)	0.09
**HCAZ at Birth**						
Maternal log iPTH	0.056 (-0.024, 0.136)	0.17	–	–	0.056 (-0.024, 0.136)	0.17
Maternal log 25(OH)D	–	–	0.016 (-0.027, 0.059)	0.46	0.016 (-0.027, 0.059)	0.46
Vitamin D Supplementation						
Placebo	–	–	ref	ref	ref	ref
4200 IU/week	–	–	-0.016 (-0.080, 0.047)	0.61	-0.016 (-0.080, 0.047)	0.61
16800 IU/week	–	–	-0.021 (-0.108, 0.067)	0.65	-0.021 (-0.108, 0.067)	0.65
28000 IU/week	–	–	-0.067 (-0.149, 0.016)	0.11	-0.067 (-0.149, 0.016)	0.11
Maternal log FGF23	0.170 (0.089, 0.250)	<0.001	0.006 (-0.004, 0.016)	0.23	0.176 (0.096, 0.256)	<0.001
Maternal log Magnesium	0.066 (-0.014, 0.145)	0.11	0.003 (-0.003, 0.009)	0.37	0.069 (-0.011, 0.148)	0.09
Estimated protein intake	-0.105 (-0.190, -0.021)	0.02	-0.016 (-0.037, 0.005)	0.14	-0.121 (-0.207, -0.035)	0.006
Season						
Spring	ref	ref	ref	ref	ref	ref
Summer	0.051 (-0.203, 0.305)	0.69	-0.036 (-0.094, 0.021)	0.22	0.015 (-0.245, 0.275)	0.91
Fall	0.104 (-0.135, 0.344)	0.39	-0.004 (-0.059, 0.051)	0.87	0.100 (-0.145, 0.344)	0.42
Winter	-0.069 (-0.319, 0.180)	0.59	-0.029 (-0.085, 0.027)	0.31	-0.098 (-0.353, 0.157)	0.45
Maternal Age	-0.025 (-0.130, 0.080)	0.64	-0.007 (-0.031, 0.017)	0.58	-0.032 (-0.139, 0.075)	0.56
Maternal Height	0.122 (0.041, 0.204)	0.003	-0.009 (-0.028, 0.010)	0.33	0.113 (0.029, 0.196)	0.008
Gravidity	0.058 (-0.045, 0.162)	0.27	-0.001 (-0.024, 0.022)	0.93	0.057 (-0.048, 0.163)	0.29
Gestational Age at Birth	-0.087 (-0.168, -0.005)	0.036	-0.014 (-0.032, 0.005)	0.16	-0.101 (-0.184, -0.017)	0.018
Asset Index	0.074 (-0.008, 0.156)	0.08	0.019 (-0.001, 0.039)	0.06	0.093 (0.009, 0.176)	0.030

[a] Direct, indirect and total effects denote the standardized effect size. Effect estimates represent the standard deviation difference in the dependent variable for every 1 standard deviation increase in the predictor variable for continuous variables and the standard deviation difference in the dependent variable within each stratum compared to the reference group for categorical variables.

[b] P<0.05 considered significant.
