# Supplementary material for: Higher maternal parathyroid hormone concentration at delivery is not associated with smaller newborn size
Source: Endocr Connect. 2021 Feb 23;10(3):345–57. doi: 10.1530/EC-21-0056 (PMC8052570; doi:10.1530/EC-21-0056)
Supplement: Supplementary Table 13. Standardized direct and indirect effects of the predictors of whole PTH (wPTH), birth length-for-age z-score (LAZ), birth weight-for-age z-score (WAZ), and birth head circumference-for-age z-score (HCAZ) (n=473)a [file supplementary_table_13.pdf]

Supplementary Table 13. Standardized direct and indirect effects of the predictors of whole PTH (wPTH), birth length-for-age z-score (LAZ), birth weight-for-age z-score (WAZ), and birth head circumference-for-age z-score (HCAZ) (n=473)[a].

	Direct Effect (95% CI)	p[b]	Indirect Effect (95% CI)	p[b]	Total Effect (95% CI)	p[b]
**Maternal log wPTH**						
Maternal log 25(OH)D	-0.182 (-0.354, -0.009)	0.039	0.013 (-0.024, 0.050)	0.49	-0.169 (-0.345, 0.007)	0.06
Vitamin D Supplementation						
Placebo	ref	ref	ref	ref	ref	ref
4200 IU/week	0.021 (-0.364, 0.407)	0.91	-0.303 (-0.585, -0.021)	0.035	-0.281 (-0.559, -0.004)	0.047
16800 IU/week	-0.251 (-0.707, 0.206)	0.28	-0.392 (-0.772, -0.012)	0.043	-0.643 (-0.909, -0.376)	<0.001
28000 IU/week	-0.286 (-0.751, 0.178)	0.23	-0.471 (-0.877, -0.066)	0.023	-0.758 (-0.997, -0.518)	<0.001
Maternal log FGF23	0.165 (0.080, 0.249)	<0.001	–	–	0.165 (0.080, 0.249)	<0.001
Maternal log Magnesium	0.107 (0.022, 0.191)	0.013	–	–	0.107 (0.022, 0.191)	0.013
Estimated protein intake	0.001 (-0.089, 0.090)	0.99	-0.013 (-0.034, 0.008)	0.23	-0.012 (-0.103, 0.079)	0.80
Season						
Spring	ref	ref	ref	ref	ref	ref
Summer	0.137 (-0.132, 0.406)	0.32	-0.032 (-0.095, 0.030)	0.31	0.105 (-0.171, 0.380)	0.46
Fall	0.215 (-0.037, 0.466)	0.09	0.026 (-0.034, 0.085)	0.40	0.240 (-0.017, 0.498)	0.07
Winter	0.308 (0.047, 0.569)	0.021	-0.018 (-0.079, 0.044)	0.57	0.290 (0.023, 0.558)	0.033
Maternal Age	-0.051 (-0.162, 0.060)	0.37	-0.002 (-0.028, 0.023)	0.85	-0.053 (-0.167, 0.060)	0.36
Maternal Height	-0.060 (-0.146, 0.026)	0.17	-0.007 (-0.027, 0.013)	0.49	-0.067 (-0.155, 0.021)	0.14
Gravidity	0.017 (-0.092, 0.126)	0.76	-0.002 (-0.027, 0.023)	0.86	0.015 (-0.097, 0.127)	0.80
Gestational Age at Birth	-0.037 (-0.124, 0.050)	0.40	-0.016 (-0.038, 0.005)	0.14	-0.053 (-0.142, 0.035)	0.24
Asset Index	0.040 (-0.047, 0.126)	0.37	0.013 (-0.008, 0.034)	0.22	0.053 (-0.036, 0.141)	0.24
**LAZ at Birth**						
Maternal log wPTH	0.031 (-0.052, 0.113)	0.47	–	–	0.031 (-0.052, 0.113)	0.47
Maternal log 25(OH)D	–	–	0.013 (-0.031, 0.058)	0.55	0.013 (-0.031, 0.058)	0.55
Vitamin D Supplementation						
Placebo	–	–	ref	ref	ref	ref
4200 IU/week	–	–	-0.008 (-0.076, 0.061)	0.83	-0.008 (-0.076, 0.061)	0.83
16800 IU/week	–	–	0.025 (-0.059, 0.108)	0.56	0.025 (-0.059, 0.108)	0.56
28000 IU/week	–	–	-0.010 (-0.095, 0.075)	0.82	-0.010 (-0.095, 0.075)	0.82
Maternal log FGF23	0.203 (0.121, 0.285)	<0.001	0.005 (-0.009, 0.019)	0.46	0.208 (0.127, 0.289)	<0.001
Maternal log Magnesium	-0.080 (-0.161, 0.002)	0.05	0.003 (-0.006, 0.012)	0.49	-0.076 (-0.157, 0.004)	0.06
Estimated protein intake	-0.104 (-0.190, -0.019)	0.017	-0.025 (-0.048, -0.002)	0.032	-0.129 (-0.217, -0.042)	0.004
Season						
Spring	ref	ref	ref	ref	ref	ref
Summer	-0.210 (-0.468, 0.048)	0.11	-0.010 (-0.075, 0.056)	0.78	-0.219 (-0.485, 0.046)	0.11
Fall	-0.120 (-0.361, 0.121)	0.33	0.014 (-0.049, 0.077)	0.66	-0.106 (-0.354, 0.142)	0.40
Winter	-0.312 (-0.563, -0.060)	0.015	-0.004 (-0.073, 0.065)	0.91	-0.316 (-0.573, -0.058)	0.02
Maternal Age	0.024 (-0.082, 0.130)	0.66	-0.006 (-0.033, 0.021)	0.67	0.018 (-0.091, 0.127)	0.74
Maternal Height	0.294 (0.212, 0.377)	<0.001	-0.009 (-0.031, 0.012)	0.40	0.285 (0.200, 0.370)	<0.001
Gravidity	0.045 (-0.059, 0.149)	0.40	-0.003 (-0.029, 0.023)	0.82	0.042 (-0.065, 0.149)	0.44
Gestational Age at Birth	-0.055 (-0.138, 0.027)	0.19	-0.015 (-0.037, 0.006)	0.16	-0.071 (-0.156, 0.014)	0.10
Asset Index	0.008 (-0.074, 0.091)	0.84	0.013 (-0.009, 0.035)	0.24	0.021 (-0.064, 0.106)	0.62
**WAZ at Birth**						
Maternal log wPTH	0.069 (0.000, 0.137)	0.05	–	–	0.069 (0.000, 0.137)	0.05
Maternal log 25(OH)D	–	–	0.004 (-0.035, 0.043)	0.85	0.004 (-0.035, 0.043)	0.85
Vitamin D Supplementation						
Placebo	–	–	ref	ref	ref	ref
4200 IU/week	–	–	-0.019 (-0.080, 0.041)	0.53	-0.019 (-0.080, 0.041)	0.53
16800 IU/week	–	–	-0.009 (-0.081, 0.063)	0.80	-0.009 (-0.081, 0.063)	0.80
28000 IU/week	–	–	-0.045 (-0.118, 0.028)	0.23	-0.045 (-0.118, 0.028)	0.23
Maternal log FGF23	0.170 (0.102, 0.238)	<0.001	0.011 (-0.001, 0.024)	0.08	0.181 (0.114, 0.249)	<0.001
Maternal log Magnesium	-0.055 (-0.123, 0.012)	0.11	0.007 (-0.002, 0.017)	0.13	-0.048 (-0.115, 0.019)	0.16
Estimated protein intake	-0.134 (-0.205, -0.063)	<0.001	-0.021 (-0.042, -0.001)	0.041	-0.155 (-0.228, -0.082)	<0.001

	Direct Effect (95% CI)	p[b]	Indirect Effect (95% CI)	p[b]	Total Effect (95% CI)	p[b]
Season						
Spring	ref	ref	ref	ref	ref	ref
Summer	-0.146 (-0.361, 0.069)	0.18	-0.004 (-0.062, 0.054)	0.88	-0.151 (-0.372, 0.071)	0.18
Fall	-0.077 (-0.278, 0.124)	0.45	0.022 (-0.033, 0.078)	0.43	-0.055 (-0.262, 0.152)	0.60
Winter	-0.197 (-0.407, 0.012)	0.07	0.008 (-0.052, 0.068)	0.80	-0.189 (-0.405, 0.026)	0.09
Maternal Age	0.013 (-0.075, 0.101)	0.78	-0.007 (-0.031, 0.016)	0.55	0.005 (-0.086, 0.097)	0.91
Maternal Height	0.146 (0.077, 0.214)	<0.001	-0.011 (-0.029, 0.008)	0.27	0.135 (0.064, 0.206)	<0.001
Gravidity	0.075 (-0.012, 0.162)	0.09	-0.002 (-0.025, 0.021)	0.88	0.073 (-0.017, 0.163)	0.11
Gestational Age at Birth	-0.196 (-0.265, -0.128)	<0.001	-0.015 (-0.034, 0.004)	0.12	-0.212 (-0.282, -0.141)	<0.001
Asset Index	0.046 (-0.023, 0.115)	0.19	0.014 (-0.006, 0.033)	0.17	0.059 (-0.012, 0.130)	0.10
**HCAZ at Birth**						
Maternal log wPTH	0.080 (-0.001, 0.162)	0.05	–	–	0.080 (-0.001, 0.162)	0.05
Maternal log 25(OH)D	–	–	0.000 (-0.040, 0.041)	0.99	0.000 (-0.040, 0.041)	0.99
Vitamin D Supplementation						
Placebo	–	–	ref	ref	ref	ref
4200 IU/week	–	–	-0.031 (-0.094, 0.032)	0.34	-0.031 (-0.094, 0.032)	0.34
16800 IU/week	–	–	-0.036 (-0.115, 0.042)	0.37	-0.036 (-0.115, 0.042)	0.37
28000 IU/week	–	–	-0.089 (-0.170, -0.008)	0.031	-0.089 (-0.170, -0.008)	0.031
Maternal log FGF23	0.168 (0.087, 0.248)	<0.001	0.013 (-0.002, 0.028)	0.08	0.181 (0.101, 0.261)	<0.001
Maternal log Magnesium	0.046 (-0.034, 0.126)	0.26	0.009 (-0.002, 0.020)	0.13	0.054 (-0.025, 0.134)	0.18
Estimated protein intake	-0.105 (-0.189, -0.020)	0.015	-0.017 (-0.038, 0.005)	0.13	-0.121 (-0.207, -0.035)	0.006
Season						
Spring	ref	ref	ref	ref	ref	ref
Summer	0.002 (-0.252, 0.256)	0.99	-0.011 (-0.071, 0.049)	0.73	-0.009 (-0.269, 0.252)	0.95
Fall	0.08 (-0.157, 0.318)	0.51	0.026 (-0.031, 0.084)	0.37	0.107 (-0.136, 0.350)	0.39
Winter	-0.094 (-0.342, 0.154)	0.46	0.001 (-0.062, 0.064)	0.98	-0.093 (-0.346, 0.160)	0.47
Maternal Age	-0.035 (-0.140, 0.069)	0.51	-0.009 (-0.034, 0.015)	0.45	-0.045 (-0.152, 0.062)	0.41
Maternal Height	0.124 (0.042, 0.205)	0.003	-0.010 (-0.030, 0.009)	0.31	0.113 (0.030, 0.197)	0.008
Gravidity	0.053 (-0.050, 0.155)	0.31	-0.001 (-0.025, 0.022)	0.91	0.051 (-0.054, 0.157)	0.34
Gestational Age at Birth	-0.109 (-0.190, -0.027)	0.009	-0.014 (-0.034, 0.005)	0.15	-0.123 (-0.207, -0.040)	0.004
Asset Index	0.065 (-0.017, 0.147)	0.12	0.018 (-0.002, 0.038)	0.08	0.083 (0.000, 0.166)	0.05

[a] Direct, indirect and total effects denote the standardized effect size. Effect estimates represent the standard deviation difference in the dependent variable for every 1 standard deviation increase in the predictor variable for continuous variables and the standard deviation difference in the dependent variable within each stratum compared to the reference group for categorical variables.

[b] P<0.05 considered significant.
