# Supplementary material for: Higher maternal parathyroid hormone concentration at delivery is not associated with smaller newborn size
Source: Endocr Connect. 2021 Feb 23;10(3):345–57. doi: 10.1530/EC-21-0056 (PMC8052570; doi:10.1530/EC-21-0056)
Supplement: Supplementary Figure 1. Selection criteria for samples eligible for analysis based on the availability of infant samples. [file supplementary_figure_1.pdf]

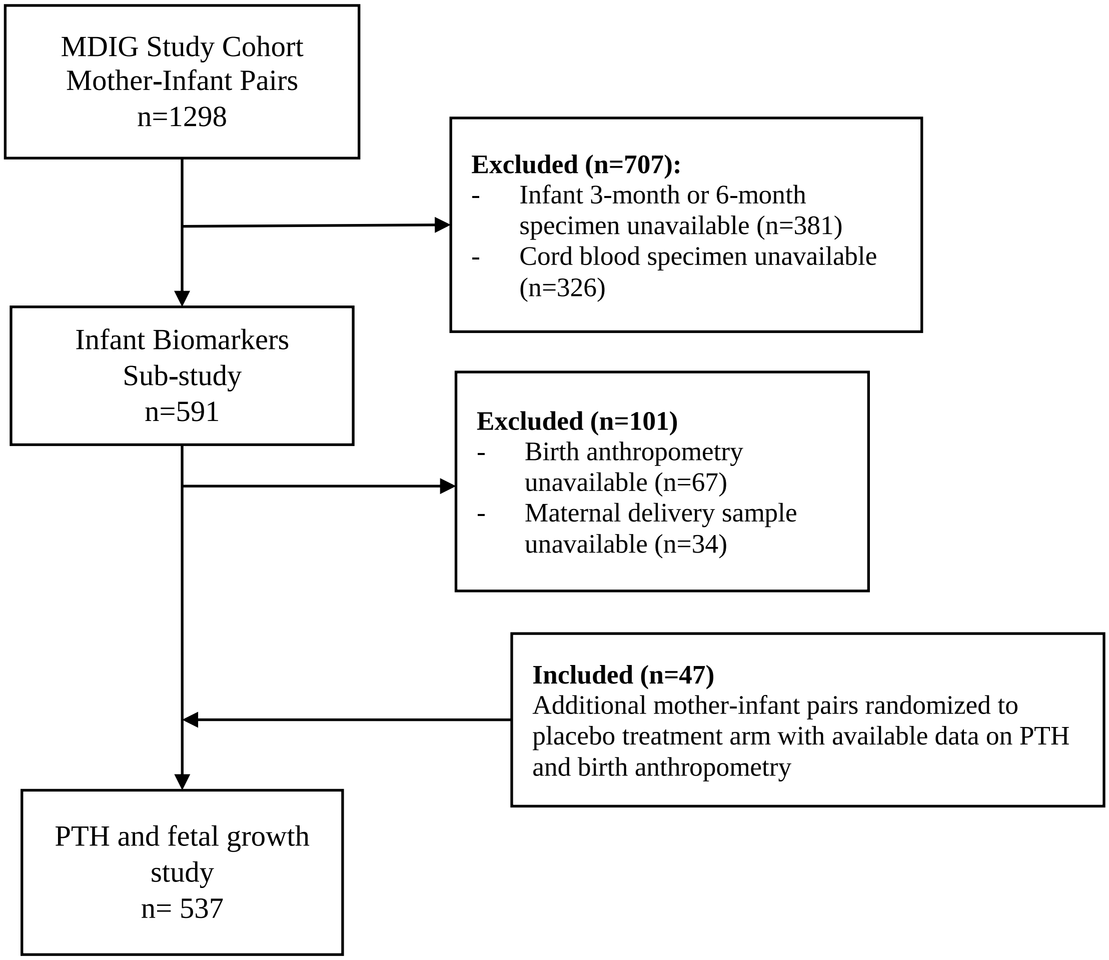

Supplementary Figure 1. Selection criteria for samples eligible for analysis based on the availability of infant samples.
