# Supplementary material for: Higher maternal parathyroid hormone concentration at delivery is not associated with smaller newborn size
Source: Endocr Connect. 2021 Feb 23;10(3):345–57. doi: 10.1530/EC-21-0056 (PMC8052570; doi:10.1530/EC-21-0056)
Supplement: Supplementary Figure 2. Comparison of PTH concentrations at mid-gestation and delivery among women randomized to the placebo group. A) Maternal iPTH concentrations (n=142) at mid-gestation (geometric mean: 3.44 pmol/L [95% CI: 3.04, 3.90]) and delivery (geometric mean: 4.9 pmol/L [95% CI: 4.35, 5.46 [file supplementary_figure_2.pdf]

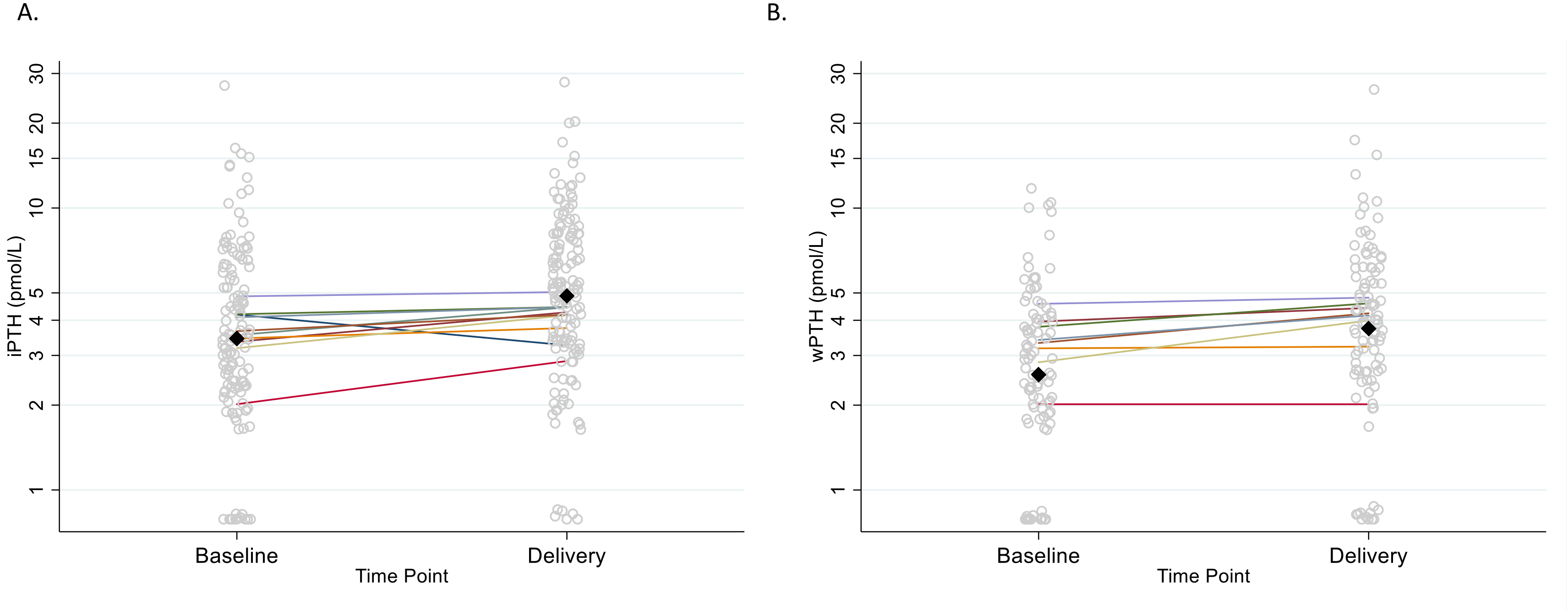

Supplementary Figure 2. Comparison of PTH concentrations at mid-gestation and delivery among women randomized to the placebo group. A) Maternal iPTH concentrations (n=142) at mid-gestation (geometric mean: 3.44 pmol/L [95% CI: 3.04, 3.90]) and delivery (geometric mean: 4.9 pmol/L [95% CI: 4.35, 5.46]); B) Maternal wPTH concentrations (n=97) at mid-gestation (geometric mean: 2.57 pmol/L [95% CI: 2.22, 2.96]) and delivery (geometric mean: 3.74 pmol/L [95% CI: 3.22, 4.35]). The black diamond denotes the geometric means. Coloured lines represent change in PTH concentrations for 10 randomly selected participants. Delivery iPTH and wPTH concentrations were both significantly higher than baseline PTH concentrations, based on paired t-tests of natural log-transformed PTH concentrations ($p<0.001$). Mid-gestation blood samples were collected between 17 and 24 weeks of gestation (median: 21 weeks), and delivery samples were collected within -19 to 4 days of delivery (median: 0 days). The cluster of data at the bottom of the graph are those women for whom iPTH or wPTH was below the respective lower limit of quantification.
