# Supplementary material for: Higher maternal parathyroid hormone concentration at delivery is not associated with smaller newborn size
Source: Endocr Connect. 2021 Feb 23;10(3):345–57. doi: 10.1530/EC-21-0056 (PMC8052570; doi:10.1530/EC-21-0056)
Supplement: Supplementary Figure 3. Lowess curves of the association between maternal iPTH and length-for-gestational age z-scores (LAZ), stratified by (A) tertiles of estimated calcium intake and (B) vitamin D supplementation. There was no significant interaction between vitamin D and iPTH and calcium intake a [file supplementary_figure_3.pdf]

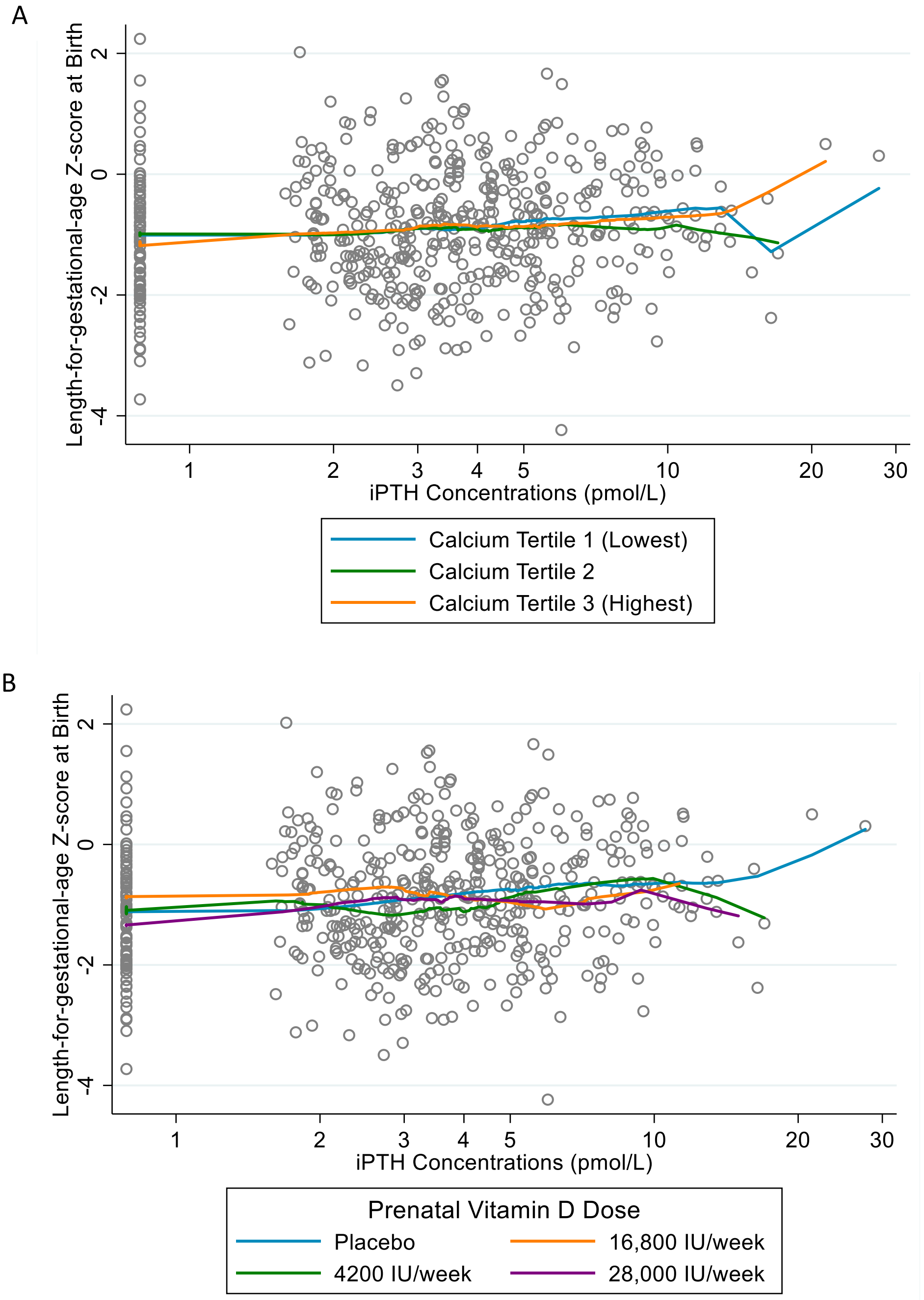

Supplementary Figure 3. Lowess curves of the association between maternal iPTH and length-for-gestational age z-scores (LAZ), stratified by (A) tertiles of estimated calcium intake and (B) vitamin D supplementation. There was no significant interaction between vitamin D and iPTH and calcium intake and iPTH ($p>0.05$) in models assessing the association between iPTH and LAZ at birth. The large concentration of points at the lower end of the iPTH distribution were below the lower limit of quantification (LOQ) and therefore, imputed as half the lower LOQ.
