# Supplementary material for: Higher maternal parathyroid hormone concentration at delivery is not associated with smaller newborn size
Source: Endocr Connect. 2021 Feb 23;10(3):345–57. doi: 10.1530/EC-21-0056 (PMC8052570; doi:10.1530/EC-21-0056)
Supplement: Supplementary Figure 4. Lowess curves of the association between maternal wPTH and weight-for-gestational age z-scores (WAZ), stratified by (A) tertiles of estimated calcium intake and (B) vitamin D supplementation. There was no significant interaction between vitamin D and wPTH and calcium intake a [file supplementary_figure_4.pdf]

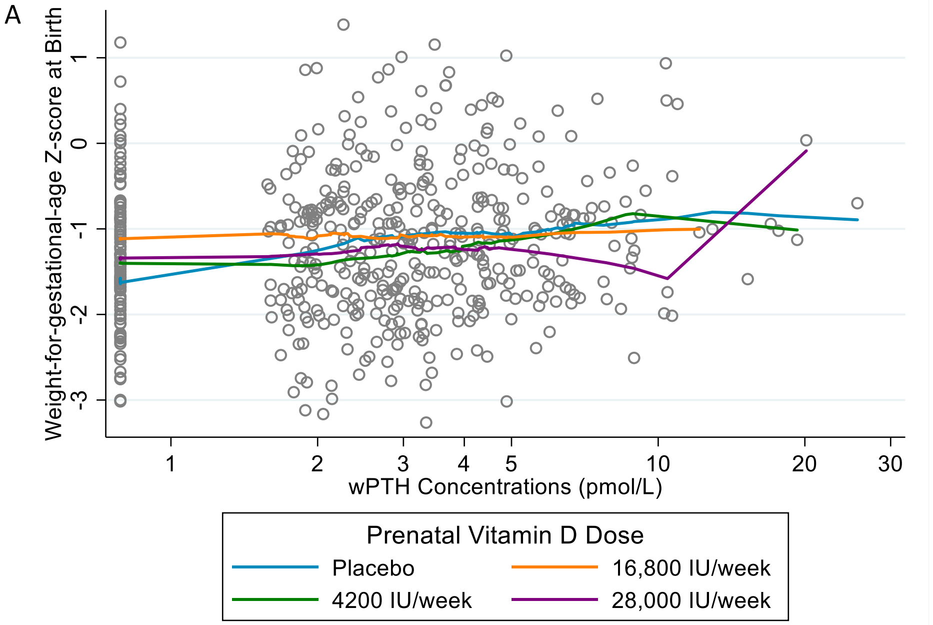

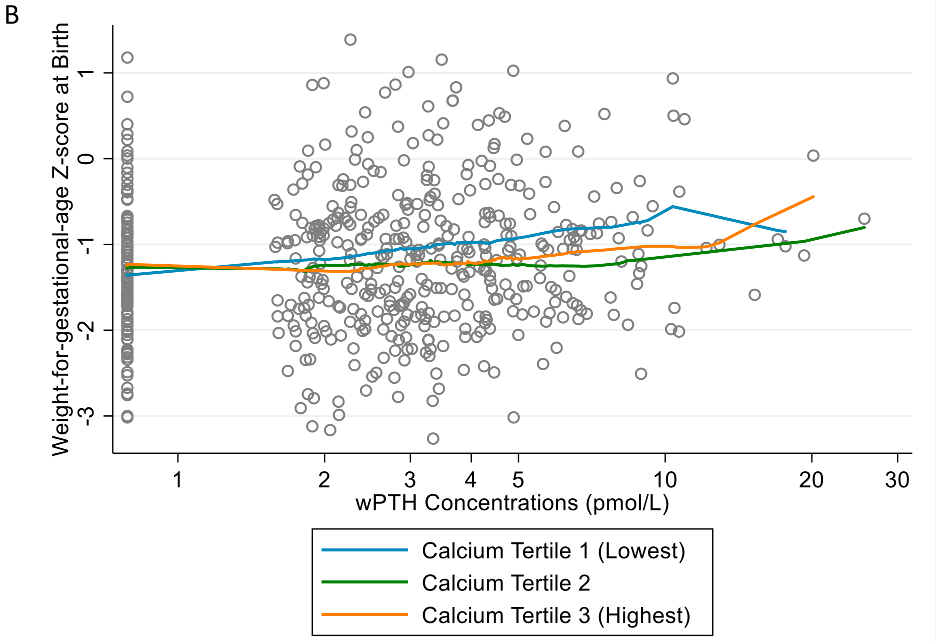

Supplementary Figure 4. Lowess curves of the association between maternal wPTH and weight-for-gestational age z-scores (WAZ), stratified by (A) tertiles of estimated calcium intake and (B) vitamin D supplementation. There was no significant interaction between vitamin D and wPTH and calcium intake and wPTH ($p>0.05$) in models assessing the association between wPTH and WAZ at birth. The large concentration of points at the lower end of the wPTH distribution were below the lower limit of quantification (LOQ) and therefore, imputed as half the lower LOQ.
